# Ecological drivers of arboviral disease risk: Vector-host interfaces in a Mediterranean wetland of Northeastern Spain

**DOI:** 10.1371/journal.pntd.0013447

**Published:** 2025-08-26

**Authors:** Júlia Froxán-Grabalosa, Simone Mariani, Catuxa Cerecedo-Iglesias, Alex Richter-Boix, Alex Ollé Torner, Magda Pla, Lluís Brotons, Frederic Bartumeus

**Affiliations:** 1 Departament d’Ecologia i Complexitat, Centre d’Estudis Avançats de Blanes (CEAB-CSIC), Blanes, Spain; 2 Servei de Control de Mosquits Badia de Roses i Baix Ter, Castelló d’Empúries, Spain; 3 Centre de Ciència i Tecnologia Forestal de Catalunya, Solsona, Spain; 4 Centre de Recerca Ecològica i Aplicacions Forestals (CREAF), Cerdanyola del Vallès, Spain; 5 CSIC, Cerdanyola del Vallès, Spain; 6 Institució Catalana de Recerca i Estudis Avançats (ICREA), Barcelona, Spain; Centers for Disease Control and Prevention, Puerto Rico, UNITED STATES OF AMERICA

## Abstract

The rising incidence of arboviral diseases poses a public health challenge worldwide. However, local-scale interactions among vectors, hosts, and the environment remain poorly understood. In this study, we analyzed historical, multi-source data to assess pathogen transmission risk in a Mediterranean wetland of Northeastern Spain, examining mosquito vectors, avian hosts for West Nile virus (WNV), and human hosts for dengue, Zika, and chikungunya. Mosquito activity peaked between June and October. *Aedes albopictus* was predominant in urban areas, whereas *Culex* species were more prevalent in rural and natural environments. The relative abundance of passeriform and columbiform bird species influenced potential amplification and dilution phenomena in the WNV enzootic cycle. We developed a spatial risk index for WNV transmission by integrating vector abundance and avian community composition. High-risk areas were identified near urban edges, particularly adjacent to rice fields and wetlands where mosquitoes and reservoir hosts overlapped. For dengue, Zika, and chikungunya, the highest transmission risk was observed in late summer, coinciding with the phenological peak of *Aedes albopictus* and the importation of cases from endemic regions. Collectively, these findings highlight the value of fine-scale ecological indicators for guiding targeted mosquito surveillance and control strategies to effectively reduce the risk of arboviral transmission in vulnerable Mediterranean regions.

## Author summary

Mosquito-borne diseases such as West Nile fever, dengue, Zika, and chikungunya, historically confined to tropical regions, are now emerging in the Mediterranean basin. Identifying where and when these diseases pose the greatest transmission risk is essential for effective public health planning. We addressed this challenge using a One Health approach that integrates multiple ecological factors: mosquito species distribution, bird community dynamics that influence virus circulation, and temporal patterns of imported human disease cases. Our analysis revealed that West Nile virus transmission risk is not uniformly distributed across the landscape. Instead, risk concentrates in natural and rural environments such as wetlands and rice fields, where mosquitoes and bird communities overlap. For dengue, Zika, and chikungunya, we identified peak transmission risk in late summer, when tourists return from endemic countries infected with these viruses. These findings have important implications for disease prevention because they demonstrate how ecological data can inform targeted public health interventions.

## Introduction

Mosquito-borne diseases have become an escalating public health concern on a global scale [1,2]. Among them, arboviruses—arthropod-borne viruses—such as dengue virus (DENV), Zika virus (ZIKV), yellow fever virus (YFV), chikungunya virus (CHIKV), and West Nile virus (WNV), are increasing in incidence and geographical distribution, both emerging in new areas and reemerging in regions from which they had previously been eradicated [3,4]. The risk of arboviral transmission is intricately linked to the interactions among arboviruses, their insect vectors, and vertebrate hosts, and is further modulated by their specific ecological and biological cycles [5].

*Aedes*-borne viruses (e.g. DENV, ZIKV, YFV, CHIKV), although zoonotic in origin and historically maintained within sylvatic cycles involving wild animals and mosquitoes, have evolved to sustain a straightforward urban transmission cycle between humans and *Aedes* mosquitoes. In this urban cycle, humans act as both primary hosts and reservoirs for these pathogens, thereby facilitating transmission and contributing to their global spread [1,6].

In contrast, WNV presents a more ecologically complex transmission cycle, primarily involving *Culex* mosquitoes and avian hosts within rural and natural environments [2,6,7]. The ecology of WNV is strongly influenced by the composition of the local bird community, as only those species capable of sustaining high viremia levels act as competent hosts, effectively facilitating viral transmission to mosquitoes [8–10]. When these competent species dominate the community, viral amplification is promoted, increasing the risk of transmission. Conversely, areas with a higher abundance of non-competent species may experience a dilution effect, reducing the likelihood of mosquito infection [8,11]. Although WNV typically circulates within this enzootic cycle between mosquitoes and birds, occasional spillover events can lead to outbreaks in urban and peri-urban settings, affecting both humans and horses. These hosts are considered dead-end hosts due to their inability to achieve sufficient viremia levels for mosquito infection; however, they remain susceptible to potentially severe disease [6].

The spread of mosquito-borne diseases is primarily facilitated by globalization and environmental change, both of which affect pathogens, vectors, and host populations [12]. The Mediterranean basin is particularly vulnerable to outbreaks due to its favorable climate, extensive tourism, and rapid land use changes near densely populated areas. Rising temperatures, prolonged summers, and increased flooding events are creating ideal conditions for mosquitoes, including urban-adapted invasive species such as *Aedes aegypti* and *Aedes albopictus* [13,14]. Notably, the expansion of *Ae. albopictus* across Europe, combined with increased human mobility from endemic regions, has created novel epidemiological scenarios that have resulted in the local transmission of dengue, Zika, and chikungunya [3].

Although urbanization favors the emergence of *Aedes*-borne diseases, land use transformation in rural and natural areas also drives transmission dynamics, particularly for pathogens such as WNV. Landscapes with extensive water bodies, such as freshwater wetlands, marshes, and rice fields, serve as ideal breeding grounds for many vectors and hosts, significantly influencing WNV ecology [3]. For instance, urban areas in proximity to rice fields, irrigated agriculture, and wetlands are considered high-risk zones for WNV human outbreaks [7]. Mediterranean countries such as Italy, Greece, and Spain exemplify this scenario, as their temperate climates, extensive agricultural activity, and the presence of wetlands and migratory bird routes create optimal conditions for WNV transmission. In recent years, Europe has witnessed a significant rise in human cases, with these three countries being among the most affected. Moreover, its geographic range has continued to expand, with 23 new regions within the European Union reporting infections for the first time in 2023 and 2024 [15,16]. This ongoing trend underscores the escalating public health threat posed by WNV across these increasingly vulnerable areas.

This study evaluates the risk of arboviral diseases in the ecologically sensitive coastal areas of northeast Spain, where local transmission of dengue and WNV has occurred sporadically since 2018 [17,18]. To proactively anticipate larger outbreaks, it is essential to understand the ecological factors driving disease circulation and amplification. To address this need, we leverage historical data from a coastal wetland area to analyze the population dynamics of key agents involved in the transmission cycles, including: (1) primary mosquito vectors (*Ae. albopictus*, *Culex pipiens*, *Culex modestus*, and *Culex theileri*), (2) avian hosts for WNV, and (3) human hosts for dengue, Zika, and chikungunya. We hypothesize that the risk of arboviral transmission is spatially and temporally heterogeneous, shaped by how different landscapes and seasonal dynamics modulate viral circulation. Focusing on a geographically well-defined area enables us to explore how local ecological conditions shape the interactions between vectors and hosts. While the analysis is region-specific, the insights gained aim to contribute to a broader understanding of transmission pathways, offering knowledge applicable to other regions with similar ecological and epidemiological contexts.

## Study area

**Fig 1:**
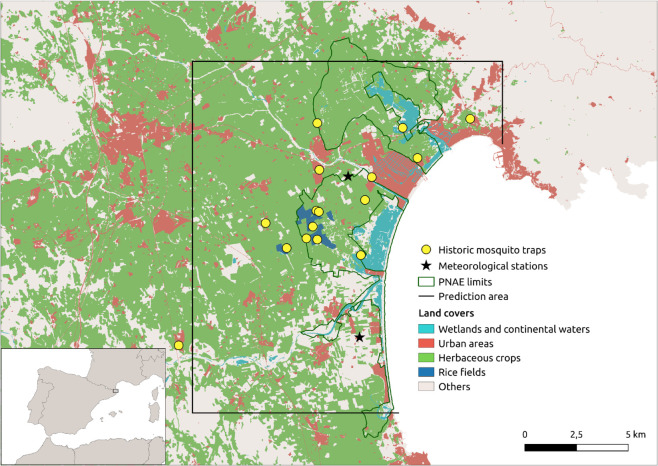
Map of the study area showing land cover types, historical mosquito surveillance sites (2001–2021), and meteorological stations. The black perimeter delimits the prediction zone, while the dark green boundary indicates the Parc Natural dels Aiguamolls de l’Empordà (PNAE). The inset map in the bottom left shows the Alt Empordà region, located in the province of Girona, Catalonia (northeastern Spain).

Parc Natural dels Aiguamolls de l’Empordà (PNAE) constitutes a major wetland ecosystem in Catalonia (northeastern Spain). Located in the Alt Empordà region of Girona province, approximately 200 km from Barcelona, it is a popular tourist destination, especially in the summer months when its population can increase fivefold. The park lies between the Fluvià and Muga river deltas, in an area characterized by fluvial systems, anthropogenic irrigation networks, rice fields and herbaceous crops (Fig 1). The PNAE represents one of Catalonia’s primary biodiversity hotspots, functioning as a crucial stopover within avian migratory corridors. The park also boasts a rich mosquito biodiversity, including several species that serve as disease vectors. Surveillance in Alt Empordà has documented WNV seropositivity in avian and equine populations [19], alongside imported cases of dengue, Zika, and chikungunya.

## Materials and methods

Statistical analyses were conducted with R version 4.3.2 [20]. Spatial distribution maps were generated with QGIS version 3.22.4-Bialowieza [21].

### Mosquito population dynamics

Entomological data were obtained from historical records owned by the Mosquito Control Service operating in the area. The monitoring network consisted of 20 trap locations deployed over a 20-year period, from 2001 to 2021, using a combination of BG traps, UV traps, and CO_2_ traps (Fig 1). However, the 20 trap locations were not continuously active throughout the entire study period; their usage varied over time, influenced by operational requirements and logistical constraints. Mosquito sampling was typically conducted over 24-hour intervals, although in certain cases, trap exposure was extended several days or even up to a week. Although these data were not collected as part of a dedicated sampling design specifically for this study, we consider them representative of the diversity of landscapes and seasonal patterns observed over the past two decades.

Over the two decades, a total of 80,886 female mosquitoes were captured. Among them, 10,270 were identified as *Ae. albopictus* and 50,052 as *Culex* spp., including 45,078 *Cx. pipiens*, 1,256 *Cx. modestus*, 2,597 *Cx. theileri*, and 1,121 *Culex* sp.. Notably, the presence of *Ae. albopictus* was first recorded in the area in 2015. To analyze the spatial and temporal variability of these species, two separate models were developed: one for *Culex* spp. and the other for *Ae. albopictus*. To enhance reliability, we consolidated the three *Culex* spp. into a single group. This decision stemmed from practical limitations in species-level identification throughout the study period. Specifically, the identification of *Cx. modestus* and *Cx. theileri* was only implemented in the later years of our study, while earlier specimens were classified exclusively as *Cx. pipiens*. This temporal inconsistency in taxonomic resolution required the pooling approach to ensure comparable data across the entire study period and provide more robust estimates of actual WNV vector presence. Moreover, this approach offers a more ecologically coherent estimate of total vector abundance relevant to WNV transmission, especially in heterogeneous environments such as rice fields, where these species co-occur.

Meteorological data were obtained from two stations within the Catalan Meteorological Network [22]: W1 (Castelló d’Empúries) and U2 (Sant Pere Pescador) (Fig 1). Station W1, located closer to the study area, served as the primary source, while station U2 provided supplementary data in cases of missing values. The dataset included daily mean (Tmean), minimum (Tmin), and maximum (Tmax) temperature, daily mean relative humidity (MRH), and daily precipitation (PPT). The integration of these variables into predictive models incorporated both daily and cumulative metrics. Cumulative values were computed as averages over 7-, 14-, and 21-day periods preceding the trap placement date, excluding the exposure period. Notably, cumulative precipitation was determined by summing values rather than averaging. Additionally, growing degree days (GDD) were calculated on both daily and cumulative bases for the same timeframes as a measure of heat accumulation, using a thermal range of 10-30^°^C suitable for mosquito development in Mediterranean climates.

Land cover data were extracted from the “2017 Land Use/Land Cover Map of Catalonia (MUCSC)”, obtained from official sources [23]. We established four main categories of interest: (1) urban areas, including roads, residential areas, and industrial zones; (2) herbaceous crops, comprising both irrigated and non-irrigated types; (3) wetlands, encompassing natural wetlands and continental water bodies due to their shared vegetation characteristics in the study area; and (4) rice fields. All other land cover types not falling into any of these primary groups were classified under a fifth category labeled as ‘Others’. We calculated the relative proportions of these four categories within a 250-meter radius influence buffer around the traps. Each trap was assigned a single predominant land cover category based on the dominant environment within its buffer. However, traps with rice fields within their buffer were directly classified as rice field-influenced, regardless of the extent of rice coverage. This adjustment was necessary because these traps are often situated along the field edges, so the buffer radius may not fully capture the influence of the rice fields on mosquito populations. Notably, this habitat classification was consistent with species-specific ecological patterns: *Ae. albopictus* was absent from rice field traps, *Cx. modestus* occurred exclusively in rice fields, *Cx. theileri* was found in rice fields and wetlands, and *Cx. pipiens* was present across all habitat types.

We employed Generalized Linear Mixed Models (GLMMs) to examine the relationship between mosquito counts and the explanatory variables. All climatic variables, including daily and cumulative measures, were included as predictors, while year, trap, and land cover variability were treated as random effects. Initially, trap type (BG, UV, CO_2_) was also included as a random effect, but model comparisons revealed it contributed negligibly to the explained variance compared to individual trap effects. Consequently, only trap identity was retained in the model, which inherently accounts for both attractant type and site-specific factors. GLMMs were computed using the lme4 package [24]. Model selection was performed with the dredge() and model.sel() functions from the MuMIn package [25], incorporating a function to assess collinearity [26]. Additionally, collinearity was evaluated using the vif() function from the car package [27], applying a threshold value of 5. Once the best combination of variables was determined for each mosquito species, we transitioned to a Bayesian hierarchical modelling framework, using the brms package [28] to develop the final models and generate predictions. The Bayesian approach employed a zero-inflated negative binomial distribution to account for the excess zeros in the mosquito count data. Priors for the model parameters were specified as Cauchy distributions with location 0 and scaling factor 2.5 [29]. To assess model fit, we used the loo package [30] to conduct leave-one-out cross-validation and evaluate predictive accuracy.

To comprehensively analyze seasonal patterns, we generated daily predictions for the period 2015–2018. This timeframe aligns with the “Atlas of Nesting Birds of Catalonia”, ensuring consistency between mosquito predictions and available avian data. We constructed seasonal curves by aggregating daily counts into weekly sums, obtaining the total predicted mosquito counts per week. From a spatial perspective, we performed point-based predictions at 150-meter intervals, incorporating land cover information at each specific coordinate. For mapping purposes, we calculated the average daily mosquito counts over the four-year period for each hypothetical trap location.

### WNV transmission dynamics in avian communities

Using a list of bird species documented in the PNAE [31], we selected 112 species, both resident and migratory, that are abundant during the mosquito season (from May to November). Spatial variability data were obtained from the “Atlas of Nesting Birds of Catalonia” [32], which provides detailed information on the distribution and abundance of selected species for the period 2015 to 2018. The Atlas data are organized using a grid system of 1×1 km squares, with each square assigned a minimum and maximum abundance value. Further details on the methodologies and data used can be found in the methodology section of the “Atlas of Nesting Birds of Catalonia” [32].

To assess WNV transmission potential, we conducted a literature review of experimental infections in avian species present within the PNAE. While some studies use field molecular and seroprevalence data to identify competent species, this approach can be problematic as it considers exposure rather than the intrinsic ability to transmit the virus [33]. In contrast, experimental studies directly measure viremia levels, which serve as a more reliable proxy for viral transmission potential. We performed a systematic search in PubMed using the following search strategy: (Bird OR Avian) AND (“West Nile virus” OR WNV) AND (experiment OR vaccine OR infection OR viremia). The resulting studies were filtered to include only those involving at least one of the 112 species identified as relevant in the PNAE. A complete list of included studies is provided in S1 Text.

To identify species with a greater potential to transmit WNV in the area, we calculated a host competence index (*H*_*comp*_) using data from experimental infections. This index quantifies the ability of a host to produce infectious mosquitoes, and is defined as the product of susceptibility (*s*), the proportion of birds that become infected as a result of exposure, infectiousness (*i*), the proportion of feeding mosquitoes that become infected after a viremic blood meal, and duration (*d*), the number of days of infectious viremia ($H_{comp}=s\;\times \;i\;\times \;d$) [10]. However, since susceptibility tends to be equal to 1, the formula is often simplified to $H_{comp}=i\;\times \;d$ [10,34]. To calculate *H*_*comp*_, we approximated *i* using viremia levels expressed in plaque-forming units (PFU)/ml, computed the daily average viremia levels for each species and subsequently calculated the area under the curve (AUC) above 10^5^ PFU/ml, the threshold required for mosquito infection [10]. This index allowed us to distinguish between reservoir species (competent in virus transmission, with an *H*_*comp*_>0) and non-reservoir species (not competent in virus transmission, with an *H*_*comp*_ = 0).

To evaluate the potential role of these bird species as WNV hosts in the study area, we calculated a host capacity index (*H*_*cap*_) using the formula $H_{cap}=H_{comp}\;\times \;I\;\times \;Ab$ [8,35,36], where *I* denotes the infection rate, and *Ab* indicates species abundance. This index estimates the number of mosquitoes that a particular species could potentially infect under hypothetical virus circulation. We obtained species abundances from the “Atlas of Nesting Birds of Catalonia” [32], selecting the maximum estimated value in each 1×1 km square and calculating a total abundance value per species for the entire study area. Given the absence of detailed seroprevalence studies and vector-host contact rate estimates in the area, we assumed a constant value for *I* and excluded this parameter from the equation. Therefore, the final formula we employed is $H_{cap}=H_{comp}\;\times \;Ab$.

Finally, we analyzed the spatial patterns of potential virus amplification and dilution by calculating the proportion of reservoir avian species (*P*_*R*_) in a 1×1 km square lattice, using the formula:


$$P_{R}=\frac{\sum Ab_{reservoirs}}{\sum Ab_{reservoirs}+\sum Ab_{non-reservoirs}}$$

### Disease risk assessment: Interactions between vectors and hosts

#### WNV risk assessment.

To generate a WNV risk index for the study area, we first up-scaled the highly spatially resolved *Culex* spp. predictions (150 m resolution) to match the scale of the bird community map (1×1 km) by averaging the predicted mosquito counts from each hypothetical trap within each of the 1×1 km grid cells. We then estimated WNV risk by multiplying these mean predicted mosquito counts (*M*) by the proportion of reservoir species (*P*_*R*_), resulting in the number of mosquitoes potentially interacting with competent avian hosts for WNV transmission ($M_{R}=M\;\times \;P_{R}$). Finally, we computed a WNV risk index by normalizing *M*_*R*_ values to a scale from 0 to 1.

Additionally, we analyzed the relationship between the WNV risk index and land use categories using a beta regression model. The WNV risk index was used as the response variable, while the land use proportions within each 1×1 km grid cell were included as explanatory variables.

#### *Aedes*-borne diseases risk assessment.

We analyzed the temporal dynamics of *Aedes*-borne disease risk by integrating three key components that drive pathogen transmission: *Ae. albopictus* population dynamics, human population seasonality, and imported case data.

To capture human population seasonality, we obtained data from the Institut d’Estadística de Catalunya [37], focusing on the Full-Time Equivalent (FTE) seasonal population metric for the Alt Empordà region. This metric accounts for population fluctuations by balancing non-resident entries and resident departures on a quarterly basis, converting individual presence to a standardized measure where one person-day equals 1/365 FTE.

For disease surveillance data, we used weekly records of imported dengue, Zika, and chikungunya cases from 2015 to 2023 for Barcelona and Girona provinces, as well as the entire Catalonia region, provided by the Public Health Agency of Catalonia (ASPCAT).

To assess transmission risk patterns, we integrated *Ae. albopictus* trap count predictions with human population seasonality and weekly averages of imported cases.

### Sensitivity analysis of WNV infection threshold

To assess the robustness of the avian WNV reservoir classification, we conducted a sensitivity analysis using an alternative 10^4^ PFU/ml infection threshold, compared to the established 10^5^ PFU/ml cutoff used in the primary analysis. By performing parallel analyses with identical methodologies for both thresholds, we assessed the impact of threshold selection on species classification and derived metrics. To further explore the implications of this alternative classification, we conducted a Principal Component Analysis (PCA) combined with k-means clustering, incorporating species abundance and host competence at the 10^4^ PFU/ml threshold.

## Results

### Mosquito population dynamics

The mosquito population models identified key climatic parameters that best explained the variability in species abundance: for *Ae. albopictus*, the 21-day cumulative maximum temperature (Tmax21), the 7-day cumulative mean relative humidity (MRH7), and the 21-day cumulative precipitation (PPT21); for *Culex* spp., the mean daytime temperature (Tmean), the 7-day cumulative mean relative humidity (MRH7), and the 21-day cumulative precipitation (PPT21) (S1 Table).

**Fig 2:**
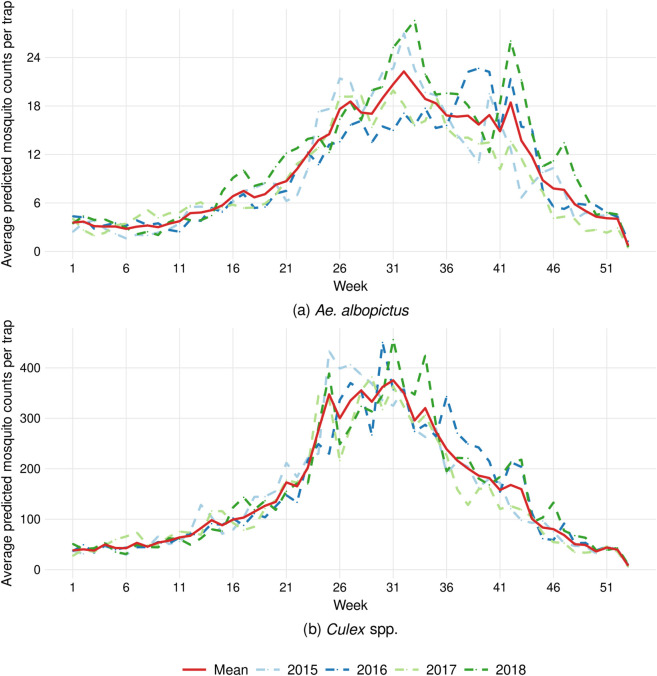
Temporal patterns of (a) *Ae. albopictus* and (b) *Culex* spp. populations from 2015 to 2018, including the mean annual variability for each species. Average predicted mosquito counts per trap are presented on a weekly basis, calculated as the sum of daily predictions.

The seasonal patterns of *Ae. albopictus* and *Culex* spp. (Fig 2) showed qualitatively similar trends but revealed remarkable differences. Trap count estimates indicated substantially higher abundance values for *Culex* spp. compared to *Ae. albopictus*. Both species reached peak abundance during the first week of August. However, *Ae. albopictus* maintained a more prolonged plateau, extending from late June to mid-October, whereas *Culex* spp. exhibited a more pronounced peak followed by a steeper decline afterwards. Raw trap count data and model predictions for both species are provided in S2 Text.

**Fig 3:**
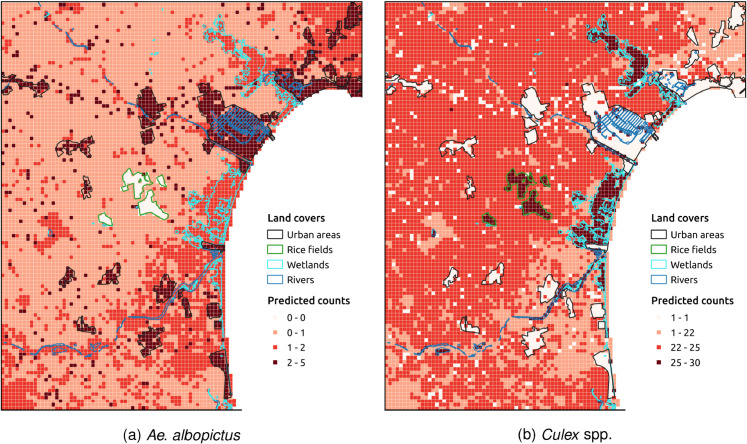
Spatial distribution of (a) *Ae. albopictus* and (b) *Culex* spp. populations. The visualization employs a color gradient based on the Natural Jenks classification method, highlighting spatial distribution patterns of both species across different land cover types. Each pixel represents the predicted number of mosquitoes that would be captured in a hypothetical trap on an average day during the period 2015–2018, at a grid resolution of 150 meters.

The average spatial distribution predictions of *Ae. albopictus* and *Culex* spp. over the same period (2015-2018) were strikingly different (Fig 3). *Ae. albopictus* predominantly occupied urban areas, with some presence around riverbeds and wetlands, and minimal presence in rice fields and agricultural areas. Conversely, *Culex* spp. thrived in natural and rural areas, showing a major presence in wetlands and rice fields, while exhibiting lower occurrence in urban settings.

### WNV transmission dynamics in avian communities

**Fig 4:**
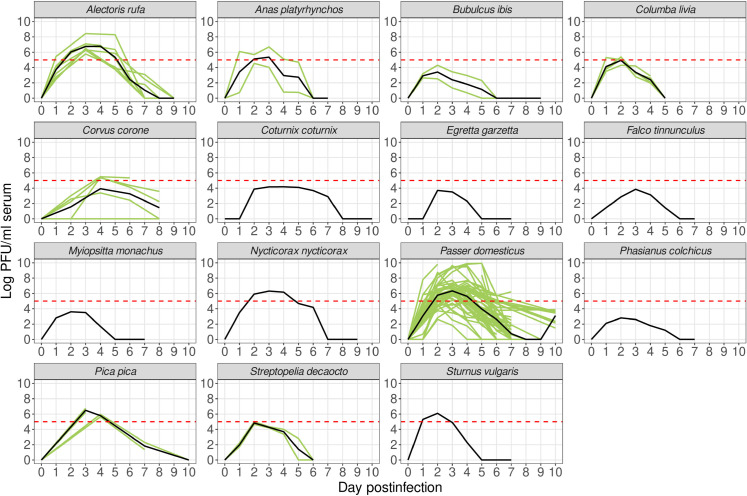
Experimental WNV viremia curves for PNAE bird species. Green lines represent individual experiment curves, while the solid black line depicts the average viremia curve. The dashed red line indicates the threshold (10^5^ PFU/ml) for mosquito infection.

Among the 112 avian species selected as most abundant in the area during the mosquito season (Table S2), we found data on WNV experimental infections for 15 species. Fig 4 shows the viremia curves for these 15 species across all reviewed experiments, with an average curve calculated for each species.

Only six of these species (*Alectoris rufa*, *Anas platyrhynchos*, *Nycticorax nycticorax*, *Passer domesticus*, *Pica pica*, and *Sturnus vulgaris*) exhibited an *H*_*comp*_>0 in our literature review (Fig 4), indicating that their average viremia curves exceed the 10^5^ PFU/ml infection threshold at some point. Consequently, these species were classified as reservoir hosts, capable of both transmitting and amplifying the virus. When considering *H*_*cap*_, which integrates both the species intrinsic competence (*H*_*comp*_) and abundance, *P. domesticus* emerged as the most significant contributor to WNV transmission in the study area, with an *H*_*cap*_ approximately 20 times greater than that of the second-ranked species (Table 1). Conversely, abundant Columbiformes in the area, such as *Columba livia* and *Streptopelia decaocto*, were classified as non-reservoir hosts (Fig 4) and thus acted as WNV dilutors within the avian community.

**Table 1:** Host competence ($H_{comp}$), abundance (*Ab*), and host capacity ($H_{cap}$) for bird species in the study area.

Species	$H_{comp}$	*Ab*	$H_{cap}$
*Passer domesticus*	2.7	15437	41679.9
*Sturnus vulgaris*	1.4	1973	2762.2
*Pica pica*	2.3	846	1945.8
*Alectoris rufa*	4.9	164	803.6
*Anas platyrhynchos*	0.5	736	368
*Nycticorax nycticorax*	3.4	100^1^	340
*Columba livia*	0	3647	0
*Streptopelia decaocto*	0	3572	0
*Myiopsitta monachus*	0	436	0
*Phasianus colchicus*	0	218	0
*Bubulcus ibis*	0	100^1^	0
*Falco tinnunculus*	0	98	0
*Coturnix coturnix*	0	80	0
*Corvus corone*	0	55	0
*Egretta garzetta*	0	2	0

*H*_*comp*_ = infectiousness (*i*) × duration (*d*).

*Ab* = $\sum_{i=1}^{n}$maximum estimated population across 1×1 km squares in the study area.

$H_{cap}=H_{comp}\;\times \;Ab$.

^1^Atlas data not available. Approximate estimate obtained from [31].

**Fig 5:**
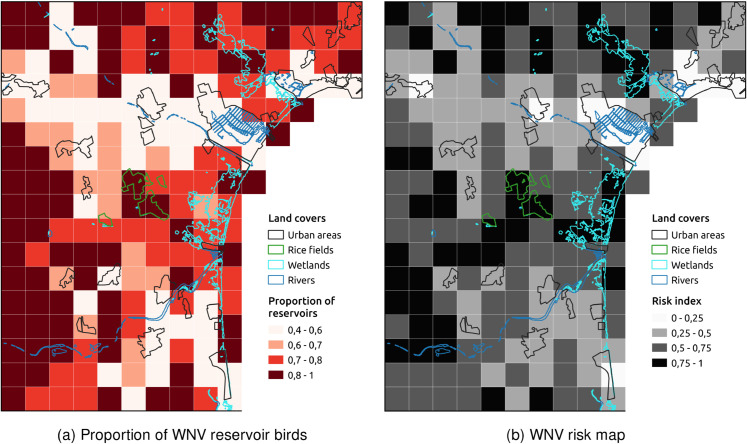
Maps of the study area, divided into 1×1 km grid cells, illustrating the proportion of WNV reservoir birds and the WNV risk index. The visualization employs an equal-interval classification scheme. **(a)** The proportion of WNV reservoir birds was calculated as the abundance of reservoir birds divided by the total abundance of reservoir and non-reservoir birds. The map represents amplification and dilution effects on WNV transmission dynamics based on the bird community composition. **(b)** The WNV risk index was calculated as the product of predicted mean *Culex* spp. mosquito counts and the proportion of WNV reservoir birds in each cell, assuming that mosquito biting patterns are proportional to host abundance. The resulting values were normalized on a scale from 0 to 1, reflecting the relative potential for mosquito-reservoir interactions. Darker areas indicate greater risk of WNV transmission due to the convergence of high vector and reservoir abundance.

Building on the identification of reservoir and non-reservoir hosts, we examined the spatial distribution of WNV amplification and dilution phenomena across the study area, considering how variations in avian community composition influence potential transmission dynamics. Fig 5(a) presents a spatial map (1 × 1 km resolution) illustrating the proportion of reservoir species relative to the total abundance of the 15 reservoir and non-reservoir species (Table 1). Cells with higher proportions, predominantly located in rice fields and agricultural areas, are likely to experience WNV amplification due to the predominance of reservoir species. In contrast, cells with lower proportions, particularly in urban areas, suggest a greater abundance of non-reservoir species, which may correspond to potential virus dilution zones.

### Disease risk assessment: Interaction between vectors and hosts

#### WNV risk assessment.

The WNV risk map, generated at 1×1 km resolution (Fig 5(b)), illustrates the spatial variation in transmission risk, with higher risk areas corresponding to regions where mosquitoes and reservoir species overlap.

Beta regression analysis examining the relationship between the WNV risk index and land cover types revealed significant associations. Urban areas showed a strong negative relationship with the risk index (β=−5.3244, $p<2\;\times \;10^{-16}$), indicating substantially lower risk in urbanized environments. In contrast, wetlands (β=3.1166, $p=3.26\;\times \;10^{-10}$) and rice fields (β=3.0331, *p* = 0.000453) were both strongly positively associated with WNV risk. No significant association was found between herbaceous crops and the risk index (β=0.2676, *p* = 0.078297).

#### *Aedes*-borne disease risk assessment.

**Fig 6:**
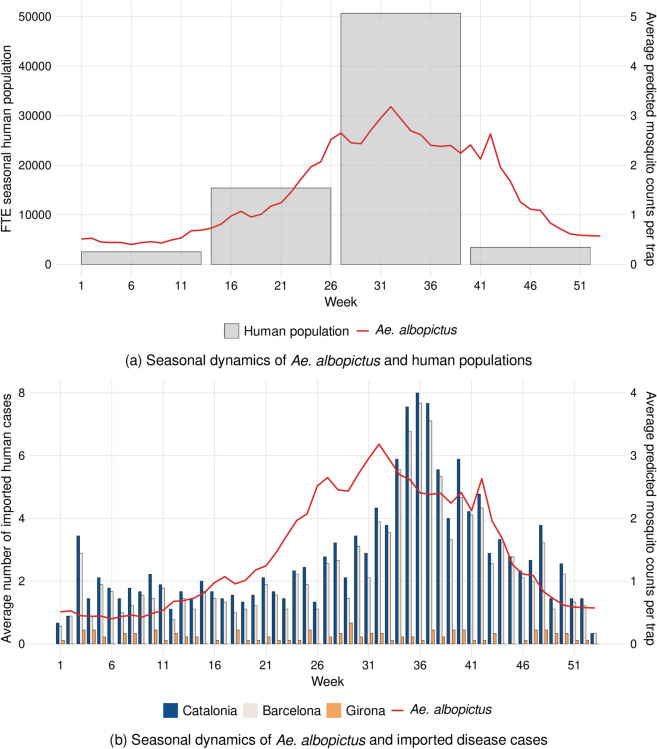
Comparison of *Ae. albopictus* seasonal dynamics with human seasonal population and imported dengue, Zika, and chikungunya cases. The red line in both panels illustrates the predicted weekly average mosquito counts per trap. **(a)** Grey bars represent the quarterly mean Full-Time Equivalent (FTE) seasonal human population in the Alt Empordà region. **(b)** Stacked bars represent the weekly average number of imported cases throughout the year, both for the entire region of Catalonia and for the provinces of Barcelona and Girona.

Our analysis revealed significant temporal synchronization between vector abundance, seasonal human population dynamics, and imported cases of dengue, Zika, and chikungunya. The integration of quarterly FTE seasonal population data with predicted *Ae. albopictus* abundance showed a pronounced concurrent peak in both metrics during July, August, and September (Fig 6(a)), emphasizing the temporal overlap of increased host and vector densities during this period.

Analysis of the temporal relationship between vector abundance and imported cases identified clear seasonal patterns (Fig 6(b)). The temporal dynamics of imported cases in Catalonia closely mirrored those observed in the province of Barcelona. The peak in imported cases occurred during week 36 (first week of September), with the highest importation period extending from week 32 to week 42. Although there was a clear correlation between vector abundance and case importation patterns, we observed a notable 4-week lag between their respective maximum peaks.

### Sensitivity analysis of WNV infection threshold

Reducing the threshold from 10^5^ to 10^4^ PFU/ml led to the reclassification of three species (*Columba livia*, *Streptopelia decaocto*, and *Coturnix coturnix*) from non-reservoirs to reservoirs (Fig A and Table A in S3 Text). Although these species now technically qualify as reservoirs, they exhibit relatively low viremia levels and brief infectious periods, indicating a limited capacity to infect mosquito vectors. Consistently, PCA and clustering analyses grouped them with non-reservoir species, indicating restricted functional competence (Fig B in S3 Text).

Spatially, lowering the threshold to 10^4^ resulted in a more homogeneous community structure across the study area. Nevertheless, WNV risk patterns remained largely unchanged, with urban areas still exhibiting lower risk due to reduced mosquito abundance (Fig C in S3 Text).

## Discussion

### Spatial and temporal dynamics of mosquito populations

Understanding arboviral transmission pathways requires an ecological perspective, which is central to the One Health approach. This framework [38] highlights the intricate interconnections between humans, disease-carrying mosquitoes, reservoir hosts, and the environments they share. Crucially, the One Health approach underscores the need to investigate mechanisms across multiple scales, identifying local environmental variability and population dynamics as key drivers of mosquito-borne disease emergence in ecologically sensitive regions.

Our analysis identified key environmental drivers shaping *Ae. albopictus* and *Culex* spp. population dynamics, revealing shared patterns and species-specific differences. Among these factors, temperature emerged as the most significant driver of population variability for both taxa, showing a positive correlation with mosquito abundance. However, previous studies have demonstrated that extreme temperatures can significantly reduce mosquito survival and reproduction [39,40]. This dual role of temperature is particularly relevant in the context of ongoing climate change: while rising temperatures may initially enhance vector abundance and accelerate viral development within mosquitoes, they may also, under extreme scenarios, push conditions beyond optimal thermal limits and disrupt phenological patterns [41]. Rainfall, in contrast, exhibited a weaker correlation, likely due to extensive water management practices in the area. Irrigation, human-mediated wetland flooding, and agricultural practices provide suitable habitats for mosquito reproduction, reducing the reliance of both *Ae. albopictus* and *Culex* spp. on natural precipitation.

Seasonal trends were similar for both taxa, with peak abundances observed between May and November, aligning with previous phenological studies conducted in Spain [42,43]. Despite these shared seasonal patterns, their spatial distributions revealed striking differences. *Ae. albopictus* is strongly associated with urban environments, relying on temporary, human-made water collections for reproduction [44,45]. In contrast, *Culex* spp. display broader ecological adaptability, thriving across a wide range of habitats, including wetlands, agricultural landscapes, and, to a lesser extent, urban areas [5,46]. Rice fields, in particular, provide ideal breeding grounds for several *Culex* spp., supporting large mosquito populations that play various roles in the transmission of mosquito-borne diseases [5].

Given that WNV transmission is sustained by multiple *Culex* mosquito species, it is essential to consider species-specific ecological roles when assessing disease dynamics. In our study, we were compelled to group several *Culex* spp., namely *Cx. pipiens*, *Cx. modestus*, and *Cx. theileri*, due to insufficient data for developing specific models for the latter two. Nonetheless, our data suggested distinct patterns among these species. *Cx. modestus*, predominantly found in rice fields, may contribute significantly to the WNV enzootic cycle due to its ornithophilic feeding behavior [47,48]. *Cx. theileri*, which also breeds in rice fields and wetlands, feeds predominantly on mammalian hosts and could have a secondary role in the epizootic cycle [47,49]. In contrast, *Cx. pipiens*, with its versatile feeding habits, is likely involved in both enzootic and epizootic cycles, potentially bridging transmission between avian and mammalian hosts [49–51]. In addition, *Cx. pipiens* was by far the most abundant mosquito species in our study area, supporting its potential leading role in local WNV transmission. This aligns with observations from other European regions, where *Cx. pipiens* consistently emerges as the primary WNV vector [52,53], although notable exceptions have been reported [54]. Finally, the anthropophilic *Ae. albopictus*, recognized as a competent vector for WNV under laboratory conditions [55,56], has also been found naturally infected in southwestern France [52], suggesting a potential involvement in urban spillover if occasional avian blood-feeding occurs.

### Amplification and dilution effects driven by avian host communities

The analysis of the avian host community yielded significant insights into species spatial variability and WNV transmission pathways. However, it also presented several limitations that, while constraining our findings, underscore important knowledge gaps. First, the bird abundance data lacked temporal variability, restricting our ability to assess how seasonal fluctuations in bird communities might influence transmission dynamics. Second, experimental WNV infection data were available for only 15 of the 112 bird species identified as ecologically relevant in the study area, limiting the scope of our analysis to this subset. Although this limited representation simplifies the broader ecological reality, it still provides a reasonable approximation for understanding how the composition of the avian community determines WNV transmission potential, as many of the reviewed species are among the most abundant in the area. Third, across these 15 species, the number of experimental studies varied considerably, resulting in greater uncertainty in viremia profiles for species with limited data. Fourth, we applied a binary classification of host infectiousness, categorizing species as competent or non-competent based on whether their mean viremia exceeded a predefined threshold. While conservative, this approach may overlook the nonlinear—often exponential—relationship between viremia and mosquito infection probability, where modest increases in viral load can lead to disproportionately higher transmission potential [57]. Finally, our analysis did not account for critical aspects of avian biology such as higher viremia levels in nestlings, brood size and timing, lifespan, population turnover, and migratory behavior, which can significantly modulate host competence and viral amplification patterns [58,59].

Despite these limitations, our analysis underscores the pivotal role of bird abundance and community composition in shaping WNV dynamics through amplification and dilution effects. These phenomena, driven by the ratio of reservoir to non-reservoir species in a given area, critically influence virus circulation and spillover potential [11,60]. In our study area, *P. domesticus* emerged as a key contributor to virus amplification due to its high host competence and widespread abundance, consistent with other European studies that have identified this species as a major amplifier of WNV transmission [61,62]. Conversely, *C. livia* and *S. decaocto*, characterized by lower competence, may primarily act as diluting agents, as suggested in previous studies [9]. Consequently, the balance between amplification and dilution is likely driven by the relative proportions of these species across different landscapes. This is particularly evident in urban areas, where the predominance of Columbiformes over *P. domesticus* likely reduces the proportion of reservoir species, thereby diminishing the overall host capacity and limiting transmission potential.

However, WNV transmission dynamics are far more complex and cannot be reduced to straightforward ratios involving only a few species. Avian communities are highly diverse, and each species likely plays a distinct role in the transmission cycle. Komar et al. (2003) [10] suggested that viremia profiles often align within phylogenetically related avian groups, allowing for broader generalizations. Passeriformes, which dominate our study area in terms of abundance, are widely recognized as the most competent group for WNV transmission [10,63]. Aside from *Passer domesticus*, other small Passeriformes, such as *Passer montanus* and *Serinus serinus*, may also contribute to virus amplification. In contrast, Columbiformes, Gruiformes, Psittaciformes, and Galliformes generally exhibit lower viremia levels [10], indicating that some abundant species within these groups may instead play a role in WNV dilution. It is worth noting, though, that the relationship between viremia and host infectiousness represents a continuous gradient rather than a strict dichotomy [33]. While we employed the 10^5^ PFU/ml threshold for classification purposes, species with viremia levels close to this value (e.g., Columbiformes) may occasionally contribute to both amplification and dilution processes, depending on individual variability, age, and vector-host interactions [33]. Even so, transmission efficiency generally becomes substantial above the 10^5^ PFU/ml threshold, supporting its use for identifying key reservoir species [10].

Adding further complexity, the capacity of each species to amplify or dilute the virus is not solely determined by its host competence and abundance, but also by mosquito feeding preferences [60,64,65]. For instance, *Cx. pipiens* has shown a distinct preference for *Turdus merula* [64], a moderately abundant species in our study area that could also play a role in WNV transmission. This hypothesis is supported by (1) the high antibody prevalence observed in Andalusia [54], (2) the species’ phylogenetic proximity to *Turdus migratorius*—a highly competent host in the United States [10,60,66]—and (3) its well-documented role as an efficient amplifier for Usutu virus in Europe [61,67]. Additionally, *Cx. pipiens* has exhibited feeding preferences for *P. pica* and *P. domesticus* [62,64], further underscoring the potential contributions of these species to local WNV amplification.

### Disease risk assessment

The analysis of vector and host populations revealed key factors influencing arboviral transmission risk. In the case of WNV, we identified rural and natural wet environments (e.g., rice fields, wetlands) as the areas of highest risk for viral amplification, where both mosquito abundance and avian community composition create optimal conditions for the enzootic cycle. This pattern is consistent with previous studies that have identified irrigated croplands and wetlands as important predictors of WNV circulation in the Mediterranean [7,46]. However, the proximity of urban centers to these areas increases the likelihood of human exposure, as both birds and mosquitoes can facilitate viral spillover into urban populations [7].

In contrast, *Aedes*-borne viruses, such as DENV, CHIKV, and ZIKV, pose a more direct threat in urban areas, where their primary vector, *Ae. albopictus*, is most prevalent. This species reaches its phenological peak between July and October, coinciding with a seasonal influx of tourists and increased pathogen importation from endemic regions. Although imported cases in Girona are few and do not exhibit a clear seasonal pattern, the strong connectivity with Barcelona, driven by secondary residences and tourist movements to northern coastal areas, likely facilitates the movement of pathogens between the two provinces. The convergence of higher vector abundance, human density, and the importation of cases from endemic areas can substantially elevate the risk of local transmission in the region [68,69].

This study enhances our understanding of the complex interactions between environmental factors, mosquito populations, avian hosts, and humans at a local scale. Our results provide valuable insights for public health management by identifying priority areas for pathogen surveillance and vector control. These results are essential not only for guiding future research efforts but also for informing public health strategies aimed at mitigating the emergence and spread of mosquito-borne diseases in the region.

### Future work

Our approach represents a foundational step in mapping arboviral risk across space and time. One of its main contributions is highlighting key data gaps and outlining a roadmap for future research to improve the accuracy and comprehensiveness of transmission models. To advance this goal, upcoming studies should examine vector and host populations at finer spatial and temporal scales, evaluating how environmental and ecological factors influence transmission dynamics. From the vector perspective, exploring *Cx. modestus* and *Cx. theileri* populations is crucial, particularly in rice fields, where these species might significantly contribute to WNV transmission. Regarding avian hosts, incorporating seasonal variability and habitat connectivity estimates based on bird movement will offer deeper insights into how population fluctuations impact transmission across diverse environments. Lastly, further research into mosquito blood-feeding preferences will provide a more comprehensive view of vector-host interactions.

## Supporting information

S1 Text References on experimental WNV infections in bird species present in the study area. (PDF)

S1 Table *Ae. albopictus* and *Culex* spp. model results. (PDF)

S2 Text Observed and predicted mosquito temporal patterns. – Fig A. *Ae. albopictus* observed and predicted temporal patterns.– Fig B. *Culex* spp. observed and predicted temporal patterns.
(PDF)

S2 Table List of bird species in the study area ranked by abundance, based on data from the “Atlas of Nesting Birds of Catalonia”, considering breeding individuals only. (PDF)

S3 TextSensitivity analysis of WNV infection threshold.– Fig A. Experimental WNV viremia curves for bird species in the study area using the 10^4^ PFU/ml threshold.– Table A. Host competence (*H*_*comp*_), abundance (*Ab*), and host capacity (*H*_*cap*_) for bird species in the study area using the 10^4^ PFU/ml threshold.– Fig B. Scatter plot illustrating the classification of avian species based on their log-transformed abundance and WNV host competence using the 10^4^ PFU/ml threshold.– Fig C. Maps of the study area, divided into 1×1 km grid cells, showing the proportion of WNV reservoir birds and the WNV risk index using the 10^4^ PFU/ml threshold.
(PDF)

## References

[1] Wilder-Smith A, Gubler DJ, Weaver SC, Monath TP, Heymann DL, Scott TW. Epidemic arboviral diseases: priorities for research and public health. Lancet Infect Dis. 2017;17(3):e101–6. doi: 10.1016/S1473-3099(16)30518-7 28011234

[2] Young PR. Arboviruses: a family on the move. In: Hilgenfeld R, Vasudevan SG, editors. Dengue and Zika: control and antiviral treatment strategies. Singapore: Springer; 2018. p. 1–10.

[3] Brugueras S, Fernández-Martínez B, Martínez-de la Puente J, Figuerola J, Porro TM, Rius C, et al. Environmental drivers, climate change and emergent diseases transmitted by mosquitoes and their vectors in southern Europe: a systematic review. Environmental Research. 2020;191:110038. doi: 10.1016/j.envres.2020.110038 32810503

[4] Franklinos LHV, Jones KE, Redding DW, Abubakar I. The effect of global change on mosquito-borne disease. Lancet Infect Dis. 2019;19(9):e302–12. doi: 10.1016/S1473-3099(19)30161-6 31227327

[5] Ferraguti M, Martínez-de la Puente J, Roiz D, Ruiz S, Soriguer R, Figuerola J. Effects of landscape anthropization on mosquito community composition and abundance. Sci Rep. 2016;6:29002. doi: 10.1038/srep29002 27373794 PMC4931447

[6] Weaver SC, Barrett ADT. Transmission cycles, host range, evolution and emergence of arboviral disease. Nat Rev Microbiol. 2004;2(10):789–801. doi: 10.1038/nrmicro1006 15378043 PMC7097645

[7] Watts MJ, Sarto I Monteys V, Mortyn PG, Kotsila P. The rise of West Nile Virus in Southern and Southeastern Europe: a spatial-temporal analysis investigating the combined effects of climate, land use and economic changes. One Health. 2021;13:100315. doi: 10.1016/j.onehlt.2021.100315 34485672 PMC8408625

[8] Johnson PTJ, Preston DL, Hoverman JT, Richgels KLD. Biodiversity decreases disease through predictable changes in host community competence. Nature. 2013;494(7436):230–3. doi: 10.1038/nature11883 23407539

[9] Kain MP, Bolker BM. Predicting West Nile virus transmission in North American bird communities using phylogenetic mixed effects models and eBird citizen science data. Parasit Vectors. 2019;12(1):395. doi: 10.1186/s13071-019-3656-8 31395085 PMC6686473

[10] Komar N, Langevin S, Hinten S, Nemeth N, Edwards E, Hettler D, et al. Experimental infection of North American birds with the New York 1999 strain of West Nile virus. Emerg Infect Dis. 2003;9(3):311–22. doi: 10.3201/eid0903.020628 12643825 PMC2958552

[11] Allan BF, Langerhans RB, Ryberg WA, Landesman WJ, Griffin NW, Katz RS, et al. Ecological correlates of risk and incidence of West Nile virus in the United States. Oecologia. 2009;158(4):699–708. doi: 10.1007/s00442-008-1169-9 18941794

[12] Semenza JC. Prototype early warning systems for vector-borne diseases in Europe. Int J Environ Res Public Health. 2015;12(6):6333–51. doi: 10.3390/ijerph120606333 26042370 PMC4483704

[13] Pardo-Araujo M, Eritja R, Alonso D, Bartumeus F. Present and future suitability of invasive and urban vectors through an environmentally driven mosquito reproduction number. Proc Biol Sci. 2024;291(2034):20241960. doi: 10.1098/rspb.2024.1960 39500373 PMC11537753

[14] Semenza JC, Suk JE. Vector-borne diseases and climate change: a European perspective. FEMS Microbiol Lett. 2018;365(2):fnx244. doi: 10.1093/femsle/fnx244 29149298 PMC5812531

[15] Carrasco L, Utrilla MJ, Fuentes-Romero B, Fernandez-Novo A, Martin-Maldonado B. West Nile Virus: an update focusing on Southern Europe. Microorganisms. 2024;12(12):2623. doi: 10.3390/microorganisms12122623 39770826 PMC11677777

[16] European Centre for Disease Prevention and Control. Surveillance of West Nile virus infections in humans and animals in Europe, monthly report. 2024. https://www.ecdc.europa.eu/en/infectious-disease-topics/west-nile-virus-infection/surveillance-and-disease-data/monthly-updates 10.2903/j.efsa.2025.9594 PMC12246792 40655556

[17] Agència de Salut Pública de Catalunya. Actualització nota informativa del dengue. 2023. https://salutpublica.gencat.cat/ca/detalls/Article/271023-dengue

[18] Agència de Salut Pública de Catalunya. Cas probable de febre del Nil Occidental a Catalunya. 2023. https://salutpublica.gencat.cat/ca/detalls/Article/230928-cas-nil-occidental

[19] Napp S, Llorente F, Beck C, Jose-Cunilleras E, Soler M, Pailler-García L, et al. Widespread circulation of flaviviruses in Horses and Birds in Northeastern Spain (Catalonia) between 2010 and 2019. Viruses. 2021;13(12):2404. doi: 10.3390/v13122404 34960673 PMC8708358

[20] R Core Team. R: A language and environment for statistical computing. R Core Team. 2021. https://www.R-project.org/

[21] QGIS Development Team. QGIS Geographic Information System. 2023. https://www.qgis.org

[22] Servei Meteorològic de Catalunya. Petició de fitxer amb dades meteorològiques; 2024. https://www.meteo.cat/wpweb/serveis/formularis/peticio-dinformes-i-dades-meteorologiques/peticio-de-dades-meteorologiques/

[23] Departament d’Agricultura, Ramaderia, Pesca i Alimentació. Bases cartogràfiques d’usos i cobertes del sòl. Departament d’Agricultura, Ramaderia, Pesca i Alimentació. 2017. https://agricultura.gencat.cat/ca/serveis/cartografia-sig/bases-cartografiques/usos-sol-subsol/usos-sol/

[24] Bates D, Mächler M, Bolker B, Walker S. Fitting linear mixed-effects models Usinglme4. J Stat Soft. 2015;67(1). doi: 10.18637/jss.v067.i01

[25] Bartoń K. MuMIn: Multi-model inference. 2024. https://CRAN.R-project.org/package=MuMIn

[26] Blanco-Sierra L, Bellver-Arnau J, Escartin S, Mariani S, Bartumeus F. Human-environment interactions shape mosquito seasonal population dynamics. Insects. 2024;15(7):527. doi: 10.3390/insects15070527 39057260 PMC11276872

[27] Fox J, Weisberg S. An R companion to applied regression. 3rd ed. California, USA: Sage Publications; 2019.

[28] Bürkner P-C. BRMS: an R package for Bayesian multilevel models using stan. J Stat Soft. 2017;80(1). doi: 10.18637/jss.v080.i01

[29] Palmer JRB, Oltra A, Collantes F, Delgado JA, Lucientes J, Delacour S, et al. Citizen science provides a reliable and scalable tool to track disease-carrying mosquitoes. Nat Commun. 2017;8(1):916. doi: 10.1038/s41467-017-00914-9 29066710 PMC5655677

[30] Vehtari A, Gabry J, Magnusson M, Yao Y, Bürkner P, Paananen T, et al. loo: efficient leave-one-out cross-validation and WAIC for Bayesian models; 2024. https://mc-stan.org/loo/.

[31] Martí-Aledo J, Ollé A. Llista dels ocells del Parc Natural dels Aiguamolls de l’Empordà (PNAE) i Badia de Roses. 2017.

[32] Franch M, Herrando S, Anton M, Villero D, Brotons L. Atles dels ocells nidificants de Catalunya: distribució i abundància 2015 –2018 i canvi des de 1980. Barcelona: Institut Català d’Ornitologia / Cossetània Edicions; 2021.

[33] Stewart Merrill TE, Johnson PTJ. Towards a mechanistic understanding of competence: a missing link in diversity-disease research. Parasitology. 2020;147(11):1159–70. doi: 10.1017/S0031182020000943 32517830 PMC10317761

[34] Komar N, Dohm DJ, Turell MJ, Spielman A. Eastern equine encephalitis virus in birds: relative competence of European starlings (Sturnus vulgaris). Am J Trop Med Hyg. 1999;60(3):387–91. doi: 10.4269/ajtmh.1999.60.387 10466964

[35] Díaz A, Flores FS, Quaglia AI, Contigiani MS. Evaluation of Argentinean Bird species as amplifying hosts for St. Louis Encephalitis Virus (Flavivirus, Flaviviridae). The American Journal of Tropical Medicine and Hygiene. 2018;99(1):216–21. doi: 10.4269/ajtmh.17-0856 29761767 PMC6085794

[36] Komar N, Panella NA, Langevin SA, Brault AC, Amador M, Edwards E, et al. Avian hosts for west nile virus in St. Tammany Parish, Louisiana 2002 . Am J Trop Med Hyg. 2005;73(6):1031–7. doi: 10.4269/ajtmh.2005.73.1031 16354808

[37] Institut d’Estadística de Catalunya. Població estacional ETCA, per trimestre; 2022. https://www.idescat.cat

[38] WHO. One Health. 2023. https://www.who.int/news-room/fact-sheets/detail/one-health

[39] Ciota AT, Matacchiero AC, Kilpatrick AM, Kramer LD. The effect of temperature on life history traits of Culex mosquitoes. J Med Entomol. 2014;51(1):55–62. doi: 10.1603/me13003 24605453 PMC3955846

[40] Delatte H, Gimonneau G, Triboire A, Fontenille D. Influence of temperature on immature development, survival, longevity, fecundity, and gonotrophic cycles of Aedes albopictus, vector of chikungunya and dengue in the Indian Ocean. J Med Entomol. 2009;46(1):33–41. doi: 10.1603/033.046.0105 19198515

[41] Mordecai EA, Caldwell JM, Grossman MK, Lippi CA, Johnson LR, Neira M, et al. Thermal biology of mosquito-borne disease. Ecol Lett. 2019;22(10):1690–708. doi: 10.1111/ele.13335 31286630 PMC6744319

[42] Casades-Martí L, Peralbo-Moreno A, Delacour-Estrella S, Ruiz-Fons F. Environmental determinants of West Nile virus vector abundance at the wildlife-livestock interface. Med Vet Entomol. 2025;39(1):200–15. doi: 10.1111/mve.12774 39499206 PMC11793132

[43] Collantes F, Méndez MJ, Soto-Castejón C, Muelas EM. Consolidation of aedes albopictus surveillance program in the autonomous community of the region of Murcia, Spain. Int J Environ Res Public Health. 2020;17(11):4173. doi: 10.3390/ijerph17114173 32545364 PMC7312822

[44] Li Y, Kamara F, Zhou G, Puthiyakunnon S, Li C, Liu Y, et al. Urbanization increases Aedes albopictus larval habitats and accelerates mosquito development and survivorship. PLoS Negl Trop Dis. 2014;8(11):e3301. doi: 10.1371/journal.pntd.0003301 25393814 PMC4230920

[45] Medlock JM, Hansford KM, Versteirt V, Cull B, Kampen H, Fontenille D, et al. An entomological review of invasive mosquitoes in Europe. Bull Entomol Res. 2015;105(6):637–63. doi: 10.1017/S0007485315000103 25804287

[46] Giesen C, Herrador Z, Fernandez-Martinez B, Figuerola J, Gangoso L, Vazquez A, et al. A systematic review of environmental factors related to WNV circulation in European and Mediterranean countries. One Health. 2023;16:100478. doi: 10.1016/j.onehlt.2022.100478 37363246 PMC10288031

[47] Muñoz J, Ruiz S, Soriguer R, Alcaide M, Viana DS, Roiz D, et al. Feeding patterns of potential West Nile virus vectors in south-west Spain. PLoS One. 2012;7(6):e39549.doi: 10.1371/journal.pone.0039549 22745781 PMC3382169

[48] Ponçon N, Balenghien T, Toty C, Baptiste Ferré J, Thomas C, Dervieux A, et al. Effects of local anthropogenic changes on potential malaria vector Anopheles hyrcanus and West Nile virus vector Culex modestus, Camargue, France. Emerg Infect Dis. 2007;13(12):1810–5. doi: 10.3201/eid1312.070730 18258028 PMC2876767

[49] Osório HC, Zé-Zé L, Alves MJ. Host-feeding patterns of Culex pipiens and other potential mosquito vectors (Diptera: Culicidae) of West Nile virus (Flaviviridae) collected in Portugal. J Med Entomol. 2012;49(3):717–21. doi: 10.1603/me11184 22679881

[50] Hamer GL, Kitron UD, Brawn JD, Loss SR, Ruiz MO, Goldberg TL, et al. Culex pipiens(Diptera: Culicidae): a bridge vector of West Nile Virus to humans. J Med Entomol. 2008;45(1):125–8. doi: 10.1093/jmedent/45.1.125 18283952

[51] Kilpatrick AM, Kramer LD, Jones MJ, Marra PP, Daszak P. West Nile virus epidemics in North America are driven by shifts in mosquito feeding behavior. PLoS Biol. 2006;4(4):e82. doi: 10.1371/journal.pbio.0040082 16494532 PMC1382011

[52] Bigeard C, Pezzi L, Klitting R, Ayhan N, L’Ambert G, Gomez N, et al. Molecular Xenomonitoring (MX) allows real-time surveillance of West Nile and Usutu virus in mosquito populations. PLoS Negl Trop Dis. 2024;18(12):e0012754. doi: 10.1371/journal.pntd.0012754 39724146 PMC11709297

[53] Mancini G, Montarsi F, Calzolari M, Capelli G, Dottori M, Ravagnan S, et al. Mosquito species involved in the circulation of West Nile and Usutu viruses in Italy. Vet Ital. 2017;53(2):97–110. doi: 10.12834/VetIt.114.933.4764.2 28675249

[54] Figuerola J, Jiménez-Clavero MÁ, Ruíz-López MJ, Llorente F, Ruiz S, Hoefer A, et al. A One Health view of the West Nile virus outbreak in Andalusia (Spain) in 2020. Emerg Microbes Infect. 2022;11(1):2570–8. doi: 10.1080/22221751.2022.2134055 36214518 PMC9621199

[55] Brustolin M, Talavera S, Santamaría C, Rivas R, Pujol N, Aranda C, et al. Culex pipiens and Stegomyia albopicta (=Aedes albopictus) populations as vectors for lineage 1 and 2 West Nile virus in Europe. Medical Vet Entomology. 2016;30(2):166–73. doi: 10.1111/mve.12164 26890285

[56] Holicki CM, Ziegler U, Răileanu C, Kampen H, Werner D, Schulz J, et al. West Nile Virus lineage 2 vector competence of indigenous culex and aedes mosquitoes from germany at temperate climate conditions. Viruses. 2020;12(5):561. doi: 10.3390/v12050561 32438619 PMC7291008

[57] Vaughan JA, Newman RA, Turell MJ. Bird species define the relationship between West Nile viremia and infectiousness to Culex pipiens mosquitoes. PLoS Negl Trop Dis. 2022;16(10):e0010835. doi: 10.1371/journal.pntd.0010835 36201566 PMC9578590

[58] Mahmood F, Chiles RE, Fang Y, Barker CM, Reisen WK. Role of nestling mourning doves and house finches as amplifying hosts of St. Louis encephalitis virus. J Med Entomol. 2004;41(5):965–72. doi: 10.1603/0022-2585-41.5.965 15535629

[59] Owen JC, Hawley DM, Huyvaert KP. Infectious disease ecology of wild birds. Oxford University Press; 2021.

[60] Kilpatrick AM, Daszak P, Jones MJ, Marra PP, Kramer LD. Host heterogeneity dominates West Nile virus transmission. Proc Biol Sci. 2006;273(1599):2327–33. doi: 10.1098/rspb.2006.3575 16928635 PMC1636093

[61] Dellar M, Sierdsema H, Schrama M, Geerling G, van Bodegom PM. The future abundance of key bird species for pathogen transmission in the Netherlands. Ecohealth. 2025:10.1007/s10393-025-01727-9.doi: 10.1007/s10393-025-01727-9 40615625 PMC12476400

[62] Rodríguez-Valencia V, Olive M-M, Le Goff G, Faisse M, Bourel M, L’Ambert G, et al. Host-feeding preferences of Culex pipiens and its potential significance for flavivirus transmission in the Camargue, France. Med Vet Entomol. 2025;39(3):614–25. doi: 10.1111/mve.12802 40118784 PMC12323745

[63] van der Meulen KM, Pensaert MB, Nauwynck HJ. West Nile virus in the vertebrate world. Arch Virol. 2005;150(4):637–57. doi: 10.1007/s00705-004-0463-z 15662484

[64] Rizzoli A, Bolzoni L, Chadwick EA, Capelli G, Montarsi F, Grisenti M, et al. Understanding West Nile virus ecology in Europe: culex pipiens host feeding preference in a hotspot of virus emergence. Parasit Vectors. 2015;8:213. doi: 10.1186/s13071-015-0831-4 25888754 PMC4411713

[65] Simpson JE, Hurtado PJ, Medlock J, Molaei G, Andreadis TG, Galvani AP, et al. Vector host-feeding preferences drive transmission of multi-host pathogens: West Nile virus as a model system. Proc Biol Sci. 2012;279(1730):925–33. doi: 10.1098/rspb.2011.1282 21849315 PMC3259921

[66] Kilpatrick AM, Dupuis AP, Chang G-JJ, Kramer LD. DNA vaccination of American robins (Turdus migratorius) against West Nile virus. Vector Borne Zoonotic Dis. 2010;10(4):377–80. doi: 10.1089/vbz.2009.0029 19874192 PMC2883478

[67] Agliani G, Visser I, Marshall EM, Giglia G, de Bruin E, Verstappen R, et al. Experimental Usutu virus infection in Eurasian blackbirds (Turdus merula). NPJ Viruses. 2025;3(1). doi: 10.1038/s44298-025-00133-w PMC12181337 40542200

[68] Barzon L. Ongoing and emerging arbovirus threats in Europe. J Clin Virol. 2018;107:38–47. doi: 10.1016/j.jcv.2018.08.007 30176404

[69] Gossner CM, Ducheyne E, Schaffner F. Increased risk for autochthonous vector-borne infections transmitted by Aedes albopictus in continental Europe. Euro Surveill. 2018;23(24):1800268. doi: 10.2807/1560-7917.ES.2018.23.24.1800268 29921345 PMC6152200

