# Supplementary material for: Ecological drivers of arboviral disease risk: Vector-host interfaces in a Mediterranean wetland of Northeastern Spain
Source: PLoS Negl Trop Dis. 2025 Aug 26;19(8):e0013447. doi: 10.1371/journal.pntd.0013447 (PMC12380343; doi:10.1371/journal.pntd.0013447)
Supplement: S1 Text — (PDF) [file pntd.0013447.s001.pdf]

References on experimental WNV infections in bird species present in the study area

1. Brault AC, Langevin SA, Ramey WN, Fang Y, Beasley DWC, Barker CM, et al. Reduced Avian Virulence and Viremia of West Nile Virus Isolates from Mexico and Texas. The American Society of Tropical Medicine and Hygiene. 2011;85:758–767. https://doi.org/10.4269/ajtmh.2011.10-0439.

2. Byas AD, Gallichotte EN, Hartwig AE, Porter SM, Gordy PW, Felix TA, et al. American alligators are capable of West Nile virus amplification, mosquito infection and transmission. Virology. 2022;568:49–55. https://doi.org/10.1016/j.virol.2022.01.009.

3. Amo JD, Llorente F, Pérez-Ramirez E, Soriguer RC, Figuerola J, Nowotny N, et al. Experimental infection of house sparrows (*Passer domesticus*) with West Nile virus strains of lineages 1 and 2. Veterinary Microbiology. 2014;172:542–547. https://doi.org/10.1016/j.vetmic.2014.06.005.

4. Amo JD, Llorente F, Figuerola J, Soriguer RC, Moreno AM, Cordioli P, et al. Experimental infection of house sparrows (*Passer domesticus*) with West Nile virus isolates of Euro-Mediterranean and North American origins. Veterinary Research. 2014;45:33. https://doi.org/10.1186/1297-9716-45-33.

5. Duggal NK, Bosco-Lauth A, Bowen RA, Wheeler SS, Reisen WK, Felix TA, et al. Evidence for Co-evolution of West Nile Virus and House Sparrows in North America. PLoS Neglected Tropical Diseases. 2014;8:e3262. https://doi.org/10.1371/journal.pntd.0003262.

6. Escribano-Romero E, de Oya NJ, Camacho MC, Blázquez AB, Martín-Acebes MA, Risalde MA, et al. Previous Usutu Virus Exposure Partially Protects Magpies (*Pica pica*) against West Nile Virus Disease But Does Not Prevent Horizontal Transmission. Viruses. 2021;13:1409. https://doi.org/10.3390/v13071409.

7. Grubaugh ND, Smith DR, Brackney DE, Bosco-Lauth AM, Fauver JR, Campbell CL, et

al. Experimental Evolution of an RNA Virus in Wild Birds: Evidence for Host-Dependent Impacts on Population Structure and Competitive Fitness. PLOS Pathogens. 2015;11:e1004874. https://doi.org/10.1371/journal.ppat.1004874.

8. Guerrero-Sánchez S, Cuevas-Romero S, Nemeth NM, Trujillo-Olivera MTJ, Worwa G, Dupuis A, et al. West Nile Virus Infection of Birds, Mexico. Emerging Infectious Diseases. 2011;17:2245–2252. https://doi.org/10.3201/eid1712.110294.

9. Jiménez de Oya N, Camacho MC, Blázquez AB, Lima-Barbero JF, Saiz JC, Höfle U, et al. High susceptibility of magpie (*Pica pica*) to experimental infection with lineage 1 and 2 West Nile virus. PLOS Neglected Tropical Diseases. 2018;12:e0006394. https://doi.org/10.1371/journal.pntd.0006394.

10. Jiménez de Oya N, Escribano-Romero E, Camacho MC, Blázquez AB, Martín-Acebes MA, Höfle U, et al. A Recombinant Subviral Particle-Based Vaccine Protects Magpie (*Pica pica*) Against West Nile Virus Infection. Frontiers in Microbiology. 2019;10. https://doi.org/10.3389/fmicb.2019.01133.

11. Kading RC, Biggerstaff BJ, Young G, Komar N. Mosquitoes Used to Draw Blood for Arbovirus Viremia Determinations in Small Vertebrates. PLoS ONE. 2014;9:e99342. https://doi.org/10.1371/journal.pone.0099342.

12. Kernbach ME, Cassone VM, Unnasch TR, Martin LB. Broad-spectrum light pollution suppresses melatonin and increases West Nile virus–induced mortality in House Sparrows (*Passer domesticus*). The Condor. 2020;122. https://doi.org/10.1093/condor/duaa018.

13. Koller KK, Kernbach ME, Reese D, Unnasch TR, Martin LB. House Sparrows Vary Seasonally in Their Ability to Transmit West Nile Virus. Physiological and Biochemical Zoology. 2023;96:332–341. https://doi.org/10.1086/725888.

14. Komar N, Langevin S, Hinten S, Nemeth N, Edwards E, Hettler D, et al. Experimental Infection of North American Birds with the New York 1999 Strain of West Nile Virus. Emerging Infectious Diseases. 2003;9:311–322. https://doi.org/10.3201/eid0903.020628.

15. Komar N, Owen JC, Edwards E, Brault AC, Langevin SA, Amador M, et al. Avian Hosts for West Nile Virus in St. Tammany Parish, Louisiana, 2002. The American Journal of Tropical Medicine and Hygiene. 2005;73:1031–1037. https://doi.org/10.4269/ajtmh.2005.73.1031.

16. Langevin SA, Bowen RA, Reisen WK, Andrade CC, Ramey WN, Maharaj PD, et al. Host Competence and Helicase Activity Differences Exhibited by West Nile Viral Variants Expressing NS3-249 Amino Acid Polymorphisms. PLoS ONE. 2014;9:e100802. https://doi.org/10.1371/journal.pone.0100802.

17. Langevin SA, C BA, Panella NA, Bown RA, Komar N. Variation in Virulence of West Nile Virus Strains for House Sparrows (*Passer domesticus*). The American Journal of Tropical Medicine and Hygiene. 2005;72:99–102. https://doi.org/10.4269/ajtmh.2005.72.99.

18. Lim SM, Brault AC, van Amerongen G, Bosco-Lauth AM, Romo H, Sewbalaksing VD, et al. Susceptibility of Carrion Crows to Experimental Infection with Lineage 1 and 2 West Nile Viruses. Emerging Infectious Diseases. 2015;21:1357–1365. https://doi.org/10.3201/eid2108.140714.

19. Llorente F, Gutiérrez-López R, Pérez-Ramirez E, Sánchez-Seco MP, Herrero L, Angel Jiménez-Clavero M, et al. Experimental infections in red-legged partridges reveal differences in host competence between West Nile and Usutu virus strains from Southern Spain. Frontiers in Cellular and Infection Microbiology. 2023;13. https://doi.org/10.3389/fcimb.2023.1163467.

20. Maharaj PD, Bosco-Lauth AM, Langevin SA, Anishchenko M, Bowen RA, Reisen WK, et al. West Nile and St. Louis encephalitis viral genetic determinants of avian host competence. PLOS Neglected Tropical Diseases. 2018;12:e0006302. https://doi.org/10.1371/journal.pntd.0006302.

21. Melian EB, Hall-Mendelin S, Du F, Owens N, Bosco-Lauth AM, Nagasaki T, et al. Programmed Ribosomal Frameshift Alters Expression of West Nile Virus Genes and Facilitates Virus Replication in Birds and Mosquitoes. PLoS Pathogens. 2014;10:e1004447. https://doi.org/10.1371/journal.ppat.1004447.

22. Molchanova EV, Prilepskaya DR, Negodenko AO, Luchinin DN, Khabarova IA. Sensitivity of Quails (*Coturnix coturnix*), Siskins (*Carduelis spinus*), and Frogs (*Rana ridibunda*) to West Nile Virus. Bulletin of Experimental Biology and Medicine. 2021;171:461–463. https://doi.org/10.1007/s10517-021-05250-z.

23. Nemeth N, Gould D, Bowen R, Komar N. Natural and Experimental West Nile Virus Infection in Five Raptor Species. Journal of Wildlife Diseases. 2006;42:1–13. https://doi.org/10.7589/0090-3558-42.1.1.

24. Nemeth NM, Oesterle PT, Bowen RA. Passive Immunity to West Nile Virus Provides Limited Protection in a Common Passerine Species. The American Journal of Tropical Medicine and Hygiene. 2008;79:283–290. https://doi.org/10.4269/ajtmh.2008.79.283.

25. Nemeth NM, Oesterle PT, Bowen RA. Humoral Immunity to West Nile Virus Is Long-Lasting and Protective in the House Sparrow (*Passer domesticus*). The American Journal of Tropical Medicine and Hygiene. 2009;80:864–869. https://doi.org/10.4269/ajtmh.2009.80.864.

26. Panella NA, Young G, Komar N. Experimental infection of Eurasian collared-dove (*Streptopelia decaocto*) with West Nile virus. Journal of Vector Ecology. 2013;38:210–214. https://doi.org/10.1111/j.1948-7134.2013.12032.x.

27. Pérez-Ramírez E, Llorente F, del Amo J, Nowotny N, Angel Jiménez-Clavero M. Susceptibility and role as competent host of the red-legged partridge after infection with lineage 1 and 2 West Nile virus isolates of Mediterranean and Central European origin. Veterinary Microbiology. 2018;222:39–45. https://doi.org/10.1016/j.vetmic.2018.06.012.

28. Reisen WK, Fang Y, Martinez VM. Avian Host and Mosquito (Diptera: Culicidae) Vector Competence Determine the Efficiency of West Nile and St. Louis Encephalitis Virus Transmission. Journal of Medical Entomology. 2005;42:367–375. https://doi.org/10.1093/jmedent/42.3.367.

29. Reisen WK, Wheeler SS, Yamamoto S, Fang Y, Garcia S. Nesting Ardeid Colonies Are Not a Focus of Elevated West Nile Virus Activity in Southern California. Vector-Borne and Zoonotic

Diseases. 2005;5:258–266. https://doi.org/10.1089/vbz.2005.5.258.

30. Sotelo E, Gutierrez-Guzmán A, Amo JD, Llorente F, El-Harrak M, Pérez-Ramírez E, et al. Pathogenicity of two recent Western Mediterranean West Nile virus isolates in a wild bird species indigenous to Southern Europe: The red-legged partridge. Veterinary Research. 2011;42. https://doi.org/10.1186/1297-9716-42-11.

31. Spedicato M, Carmine I, Bellacicco AL, Marruchella G, Marini V, Pisciella M, et al. Experimental infection of rock pigeons (*Columba livia*) with three West Nile virus lineage 1 strains isolated in Italy between 2009 and 2012. Epidemiology and Infection. 2016;144:1301–1311. https://doi.org/10.1017/S0950268815002642.

32. Wheeler SS, Barker CM, Fang Y, Armijos MV, Carroll BD, Husted S, et al. Differential Impact of West Nile Virus on California Birds. The Condor. 2009;111:1–20. https://doi.org/10.1525/cond.2009.080013.

33. Wheeler SS, Langevin SA, Brault AC, Woods L, Carroll BD, Reisen WK. Detection of Persistent West Nile Virus RNA in Experimentally and Naturally Infected Avian Hosts. The American Journal of Tropical Medicine and Hygiene. 2012;87:559–564. https://doi.org/10.4269/ajtmh.2012.11-0654.

34. Work TH, Hurlbut HS, Taylor RM. Indigenous Wild Birds of the Nile Delta as Potential West Nile Virus Circulating Reservoirs 1. The American Journal of Tropical Medicine and Hygiene. 1955;4:872–888. https://doi.org/10.4269/ajtmh.1955.4.872.
