# Supplementary material for: Ecological drivers of arboviral disease risk: Vector-host interfaces in a Mediterranean wetland of Northeastern Spain
Source: PLoS Negl Trop Dis. 2025 Aug 26;19(8):e0013447. doi: 10.1371/journal.pntd.0013447 (PMC12380343; doi:10.1371/journal.pntd.0013447)
Supplement: S1 Table — (PDF) [file pntd.0013447.s002.pdf]

Table S1. *Ae. albopictus* and *Culex* spp. model results. Land covers and interactions between Year and Trap variability were introduced as random effects, improving the goodness of fit. Covariates were all scaled.

	Estimate	SE	l-95% CI	u-95% CI
Ae. albopictus				
**Group-Level Effects**				
Land cover sd (Intercept)	3.22	1.48	1.42	6.98
Year:Trap sd (Intercept)	1.50	0.23	1.13	2.02
Population-Level Effects				
Intercept	-1.11	1.54	-4.16	2.00
scale-Tmax21	0.38	0.03	0.32	0.43
scale-MRH7	0.15	0.02	0.11	0.20
scale-PPT21	0.21	0.02	0.16	0.25
***Culex* spp.**				
**Group-Level Effects**				
Land cover sd (Intercept)	2.26	1.14	0.96	5.26
Year:Trap sd (Intercept)	1.02	0.08	0.88	1.20
**Population-Level Effects**				
Intercept	2.15	1.20	-0.35	4.38
scale-Tmean	0.46	0.03	0.41	0.51
scale-MRH7	-0.06	0.02	-0.10	-0.01
scale-PPT21	0.14	0.03	0.09	0.19

Tmax21 = Average daily maximum temperature over the 21 days prior to the trapping period. MRH7 = Average daily mean relative humidity over the 7 days prior to the trapping period. PPT21 = Cumulative daily precipitation over the 21 days prior to the trapping period. Tmean = Average daily mean temperature over the trapping period.
