# Supplementary material for: Ecological drivers of arboviral disease risk: Vector-host interfaces in a Mediterranean wetland of Northeastern Spain
Source: PLoS Negl Trop Dis. 2025 Aug 26;19(8):e0013447. doi: 10.1371/journal.pntd.0013447 (PMC12380343; doi:10.1371/journal.pntd.0013447)
Supplement: S2 Text — – Fig A. Ae. albopictus observed and predicted temporal patterns.– Fig B. Culex spp. observed and predicted temporal patterns. (PDF) [file pntd.0013447.s003.pdf]

Observed and predicted mosquito temporal patterns

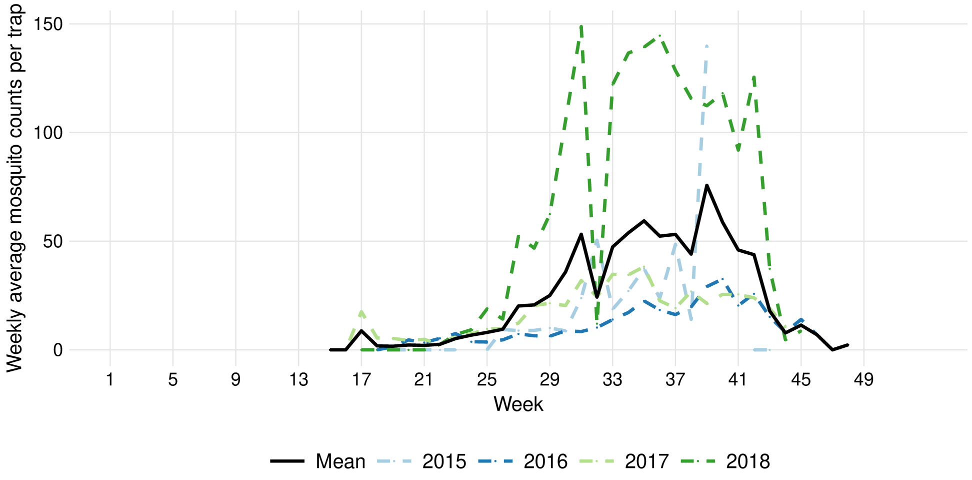

(a) *Ae. albopictus* raw data (2015-2018)

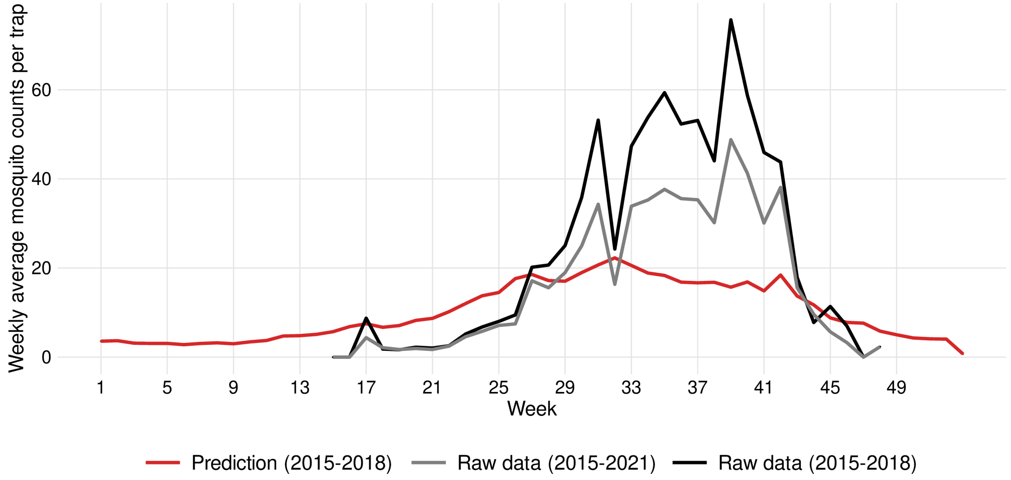

(b) *Ae. albopictus* prediction vs. raw data

Fig A. *Aedes albopictus* observed and predicted temporal patterns. **(a)** Raw observational data from 2015–2018. **(b)** Comparison of predicted counts (2015–2018) with raw data averages for the same period (2015–2018) and the full training dataset (2015–2021). The systematically lower predicted values likely reflect spatial sampling bias in the raw data, which overrepresents urban environments where *Ae. albopictus* thrives. In contrast, predictions assume a more representative landscape composition, with predominantly rural and natural areas where this species is naturally less abundant.

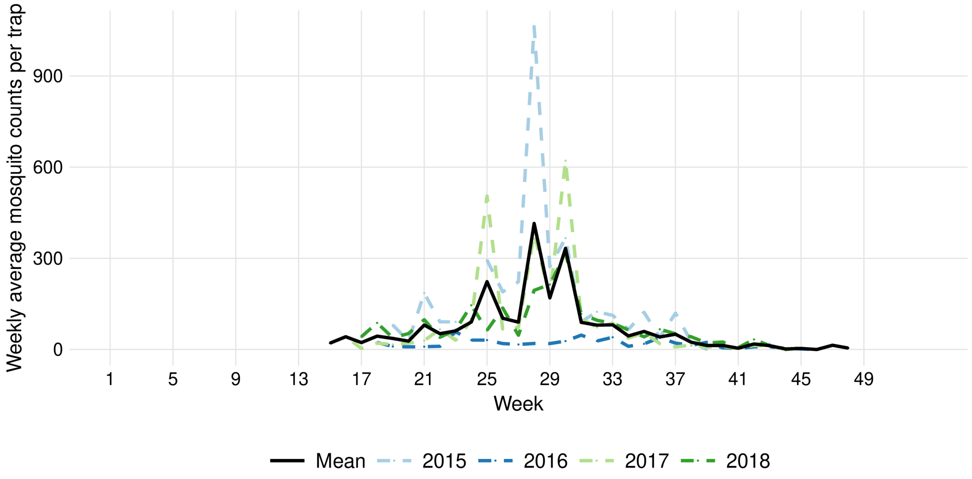

(a) *Culex* spp. raw data (2015-2018)

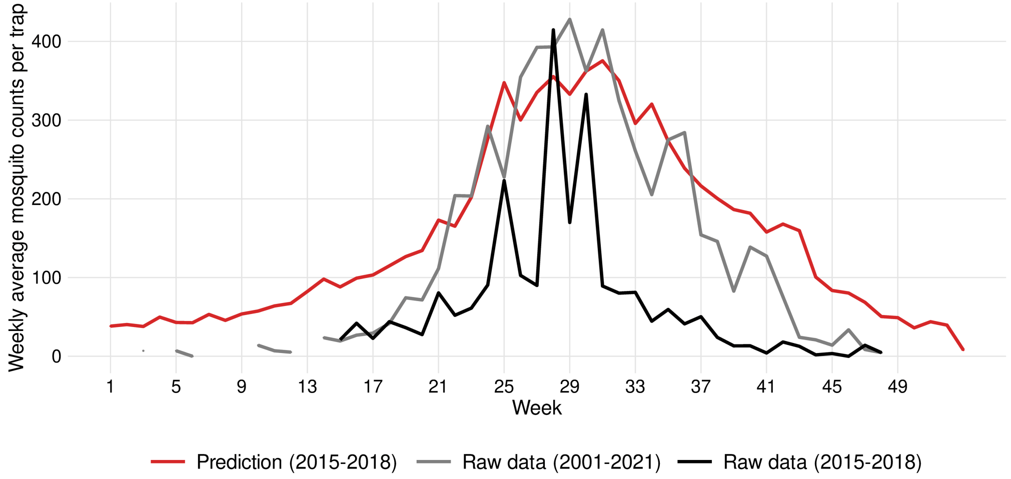

(b) *Culex* spp. prediction vs. raw data

Fig B. *Culex* spp. observed and predicted temporal patterns. **(a)** Raw observational data from 2015–2018. **(b)** Comparison of predicted counts (2015–2018) with raw data averages for the same period (2015–2018) and the full training dataset (2001–2021). The predictions closely match the average pattern observed across the complete time series (2001–2021). For the 2015–2018 period, abundance levels are similar between observed and predicted data, but seasonal patterns show some differences. These discrepancies likely reflect sampling limitations and biases inherent in the observational data, which become more pronounced over shorter temporal windows.
