# Supplementary material for: Ecological drivers of arboviral disease risk: Vector-host interfaces in a Mediterranean wetland of Northeastern Spain
Source: PLoS Negl Trop Dis. 2025 Aug 26;19(8):e0013447. doi: 10.1371/journal.pntd.0013447 (PMC12380343; doi:10.1371/journal.pntd.0013447)
Supplement: S2 Table — (PDF) [file pntd.0013447.s004.pdf]

Table S2. List of bird species in the study area ranked by abundance, based on data from the "Atlas of Nesting Birds of Catalonia", considering breeding individuals only.

Scientific Name	Family	Order	Abundance
Passer domesticus	Passeridae	Passeriformes	15437.17
Passer montanus	Passeridae	Passeriformes	8891.38
Serinus serinus	Fringillidae	Passeriformes	6729.67
Hirundo rustica	Hirundinidae	Passeriformes	5657.64
Luscinia megarhynchos	Muscicapidae	Passeriformes	5352.09
Columba livia	Columbidae	Columbiformes	3647.18
Streptopelia decaocto	Columbidae	Columbiformes	3572.20
Carduelis carduelis	Fringillidae	Passeriformes	3544.13
Cisticola juncidis	Cisticolidae	Passeriformes	2937.05
Columba palumbus	Columbidae	Columbiformes	2425.96
Chloris chloris	Fringillidae	Passeriformes	2366.60
Hippolais polyglotta	Acrocephalidae	Passeriformes	2307.40
Delichon urbicum	Hirundinidae	Passeriformes	2015.32
Sturnus vulgaris	Sturnidae	Passeriformes	1973.17
Apus apus	Apodidae	Apodiformes	1948.83
Galerida cristata	Alaudidae	Passeriformes	1820.56
Corvus monedula	Corvidae	Passeriformes	1496.07
Emberiza calandra	Emberizidae	Passeriformes	1431.92
Sylvia melanocephala	Sylviidae	Passeriformes	1402.77
Parus major	Paridae	Passeriformes	1200.61
Sylvia atricapilla	Sylviidae	Passeriformes	907.28
Cettia cetti	Cettiidae	Passeriformes	867.45
Linaria cannabina	Fringillidae	Passeriformes	855.57
Pica pica	Corvidae	Passeriformes	845.78
Larus michahellis	Laridae	Charadriiformes	773.22

Scientific Name	Family	Order	Abundance
Anas platyrhynchos	Anatidae	Anseriformes	735.74
Turdus merula	Turdidae	Passeriformes	599.54
Motacilla alba	Motacillidae	Passeriformes	492.92
Upupa epops	Upupidae	Bucerotiformes	456.25
Myiopsitta monachus	Psittacidae	Psittaciformes	436.00
Emberiza cirlus	Emberizidae	Passeriformes	369.87
Merops apiaster	Meropidae	Coraciiformes	336.14
Oriolus oriolus	Oriolidae	Passeriformes	322.38
Rallus aquaticus	Rallidae	Gruiformes	274.34
Athene noctua	Strigidae	Strigiformes	270.35
Certhia brachydactyla	Certhiidae	Passeriformes	239.63
Phasianus colchicus	Phasianidae	Galliformes	217.80
Saxicola rubicola	Muscicapidae	Passeriformes	200.53
Aegithalos caudatus	Aegithalidae	Passeriformes	195.90
Otus scops	Strigidae	Strigiformes	193.93
Apus pallidus	Apodidae	Apodiformes	183.06
Cyanistes caeruleus	Paridae	Passeriformes	176.57
Himantopus himantopus	Recurvirostridae	Charadriiformes	174.65
Alectoris rufa	Phasianidae	Galliformes	163.80
Gallinula chloropus	Rallidae	Gruiformes	162.38
Lanius senator	Laniidae	Passeriformes	158.60
Petronia petronia	Passeridae	Passeriformes	145.12
Charadrius alexandrinus	Charadriidae	Charadriiformes	139.46
Fulica atra	Rallidae	Gruiformes	136.86
Acrocephalus scirpaceus	Acrocephalidae	Passeriformes	129.77
Streptopelia turtur	Columbidae	Columbiformes	125.40
Burhinus oedicnemus	Burhinidae	Charadriiformes	109.85

Scientific Name	Family	Order	Abundance
Erithacus rubecula	Muscicapidae	Passeriformes	102.70
Falco tinnunculus	Falconidae	Falconiformes	97.52
Muscicapa striata	Muscicapidae	Passeriformes	95.84
Alauda arvensis	Alaudidae	Passeriformes	94.73
Motacilla flava	Motacillidae	Passeriformes	93.67
Coturnix coturnix	Phasianidae	Galliformes	79.95
Garrulus glandarius	Corvidae	Passeriformes	75.37
Clamator glandarius	Cuculidae	Cuculiformes	65.88
Charadrius dubius	Charadriidae	Charadriiformes	61.70
Troglodytes troglodytes	Troglodytidae	Passeriformes	60.13
Acrocephalus arundinaceus	Acrocephalidae	Passeriformes	59.11
Apus melba	Apodidae	Apodiformes	58.78
Sylvia hortensis	Sylviidae	Passeriformes	57.93
Sylvia cantillans	Sylviidae	Passeriformes	57.31
Corvus corone	Corvidae	Passeriformes	55.08
Tyto alba	Tytonidae	Strigiformes	46.98
Phoenicurus ochruros	Muscicapidae	Passeriformes	45.25
Tachybaptus ruficollis	Podicipedidae	Podicipediformes	43.92
Picus viridis	Picidae	Piciformes	41.52
Buteo buteo	Accipitridae	Accipitriformes	33.31
Asio otus	Strigidae	Strigiformes	32.51
Cuculus canorus	Cuculidae	Cuculiformes	32.03
Acrocephalus melanopogon	Acrocephalidae	Passeriformes	29.41
Alcedo atthis	Alcedinidae	Coraciiformes	23.96
Circus aeruginosus	Accipitridae	Accipitriformes	14.96
Dendrocopos major	Picidae	Piciformes	11.68
Falco subbuteo	Falconidae	Falconiformes	11.17

Scientific Name	Family	Order	Abundance
Ixobrychus minutus	Ardeidae	Pelecaniformes	8.91
Remiz pendulinus	Remizidae	Passeriformes	7.92
Ciconia ciconia	Ciconiidae	Ciconiiformes	7.54
Corvus corax	Corvidae	Passeriformes	5.58
Coracias garrulus	Coraciidae	Coraciiformes	5.34
Podiceps cristatus	Podicipedidae	Podicipediformes	4.51
Psittacula krameri	Psittaculidae	Psittaciformes	4.21
Egretta garzetta	Ardeidae	Pelecaniformes	2.17
Milvus migrans	Accipitridae	Accipitriformes	2.00
Ardea cinerea	Ardeidae	Pelecaniformes	1.99
Accipiter gentilis	Accipitridae	Accipitriformes	0.52
Falco peregrinus	Falconidae	Falconiformes	0.35
Anas crecca	Anatidae	Anseriformes	NA
Ardea purpurea	Ardeidae	Pelecaniformes	NA
Bubulcus ibis	Ardeidae	Pelecaniformes	NA
Calidris alpina	Scolopacidae	Charadriiformes	NA
Calidris minuta	Scolopacidae	Charadriiformes	NA
Calidris pugnax	Scolopacidae	Charadriiformes	NA
Charadrius hiaticula	Charadriidae	Charadriiformes	NA
Chroicocephalus ridibundus	Laridae	Charadriiformes	NA
Dryobates minor	Picidae	Piciformes	NA
Gallinago gallinago	Scolopacidae	Charadriiformes	NA
Mareca strepera	Anatidae	Anseriformes	NA
Nycticorax nycticorax	Ardeidae	Pelecaniformes	NA
Phalacrocorax carbo	Phalacrocoracidae	Suliformes	NA
Phoenicopterus roseus	Phoenicopteridae	Phoenicopteriformes	NA
Riparia riparia	Hirundinidae	Passeriformes	NA

Scientific Name	Family	Order	Abundance
Spatula clypeata	Anatidae	Anseriformes	NA
Sylvia borin	Sylviidae	Passeriformes	NA
Tringa glareola	Scolopacidae	Charadriiformes	NA
Tringa nebularia	Scolopacidae	Charadriiformes	NA
Tringa totanus	Scolopacidae	Charadriiformes	NA
Vanellus vanellus	Charadriidae	Charadriiformes	NA
