# Supplementary material for: Ecological drivers of arboviral disease risk: Vector-host interfaces in a Mediterranean wetland of Northeastern Spain
Source: PLoS Negl Trop Dis. 2025 Aug 26;19(8):e0013447. doi: 10.1371/journal.pntd.0013447 (PMC12380343; doi:10.1371/journal.pntd.0013447)
Supplement: S3 Text — – Fig A. Experimental WNV viremia curves for bird species in the study area using the 104 PFU/ml threshold.– Table A. Host competence (Hcomp), abundance (Ab), and host capacity (Hcap) for bird species in the study area using the 104 PFU/ml threshold.– Fig B. Scatter plot illustrating the classification of avian species based on their log-transformed abundance and WNV host competence using the 104 PFU/ml threshold.– Fig C. Maps of the study area, divided into 1×1 km grid cells, showing the proportion of WNV reservoir birds and the WNV risk index using the 104 PFU/ml threshold. (PDF) [file pntd.0013447.s005.pdf]

Sensitivity analysis of WNV infection threshold

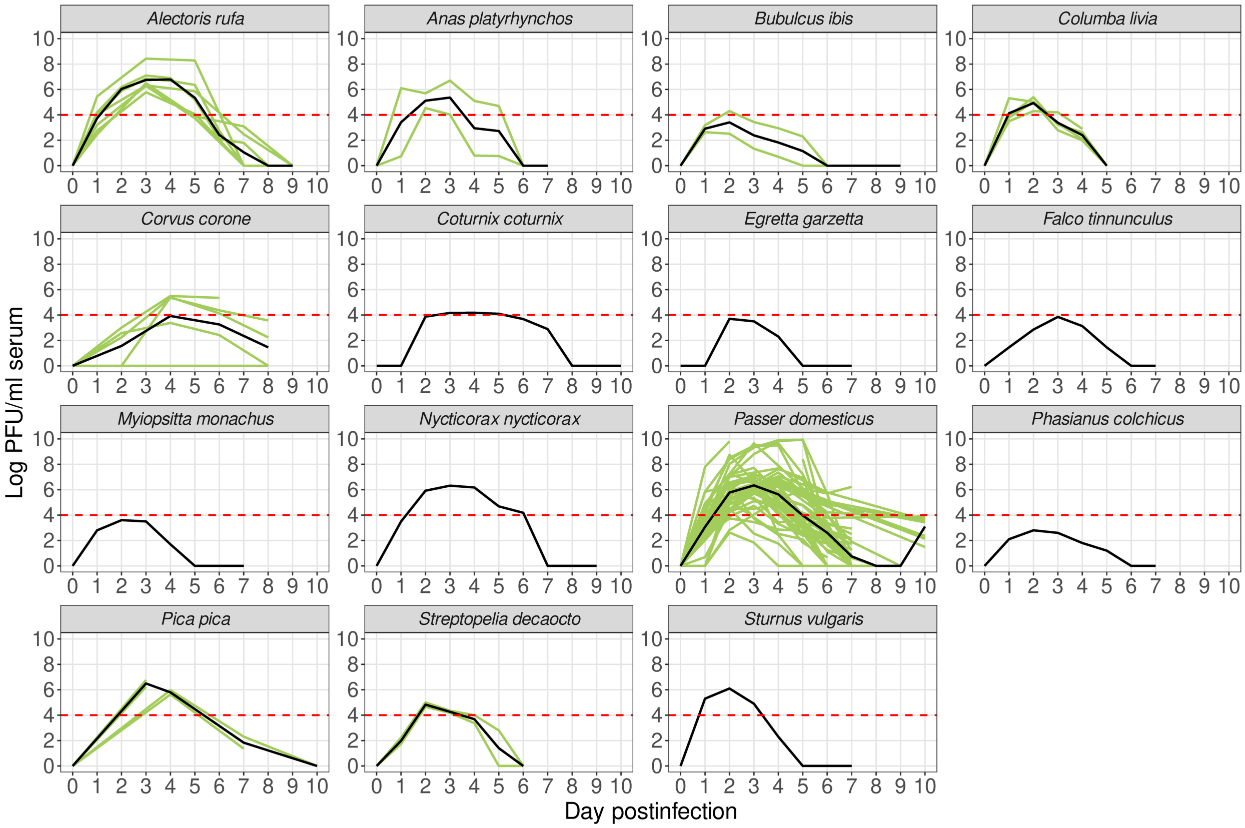

Fig A. Experimental WNV viremia curves for bird species in the study area using the 10^4 PFU/ml threshold. Green lines represent individual experiment curves, while the solid black line depicts the average viremia curve. The dashed red line indicates the 10^4 PFU/ml threshold. Compared to the primary analysis using a 10^5 PFU/ml threshold, certain species previously classified as non-reservoirs, specifically *Columba livia*, *Streptopelia decaocto*, and *Coturnix coturnix*, now exceed the infection threshold and are reclassified as potential reservoirs. However, although these species exceed the 10^4 PFU/ml threshold, their viremia levels remain in close proximity to this limit and do not persist over time, resulting in a relatively short theoretical infectious period.

Table A. Host competence (H_{comp}), abundance (Ab), and host capacity (H_{cap}) for bird species in the study area using the 10^4 PFU/ml threshold. The number of species classified as reservoirs has increased compared to the original threshold, leading to shifts in species contribution to virus amplification.

Species	H_{comp}	Ab	H_{cap}
Passer domesticus	5.7	15437	87990.9
Sturnus vulgaris	4.3	1973	8483.9
Pica pica	5.1	846	4314.6
Columba livia	1.1	3647	4011.7
Streptopelia decaocto	1.1	3572	3929.2
Anas platyrhynchos	2.5	736	1840.0
Alectoris rufa	8.9	164	1459.6
Nycticorax nycticorax	7.3	100[1]	730.0
Coturnix coturnix	0.4	80	32.0
Myiopsitta monachus	0	436	0
Phasianus colchicus	0	218	0
Bubulcus ibis	0	100[1]	0
Falco tinnunculus	0	98	0
Corvus corone	0	55	0
Egretta garzetta	0	2	0

H_{comp} = infectiousness (i) * duration (d)

$Ab = \sum_{i=1}^{n}$maximum estimated population across 1x1 km squares in the study area

$H_{cap} = H_{comp} * Ab$

[1]Atlas data not available. Approximate estimate obtained from Martí-Aledo and Ollé (2017).

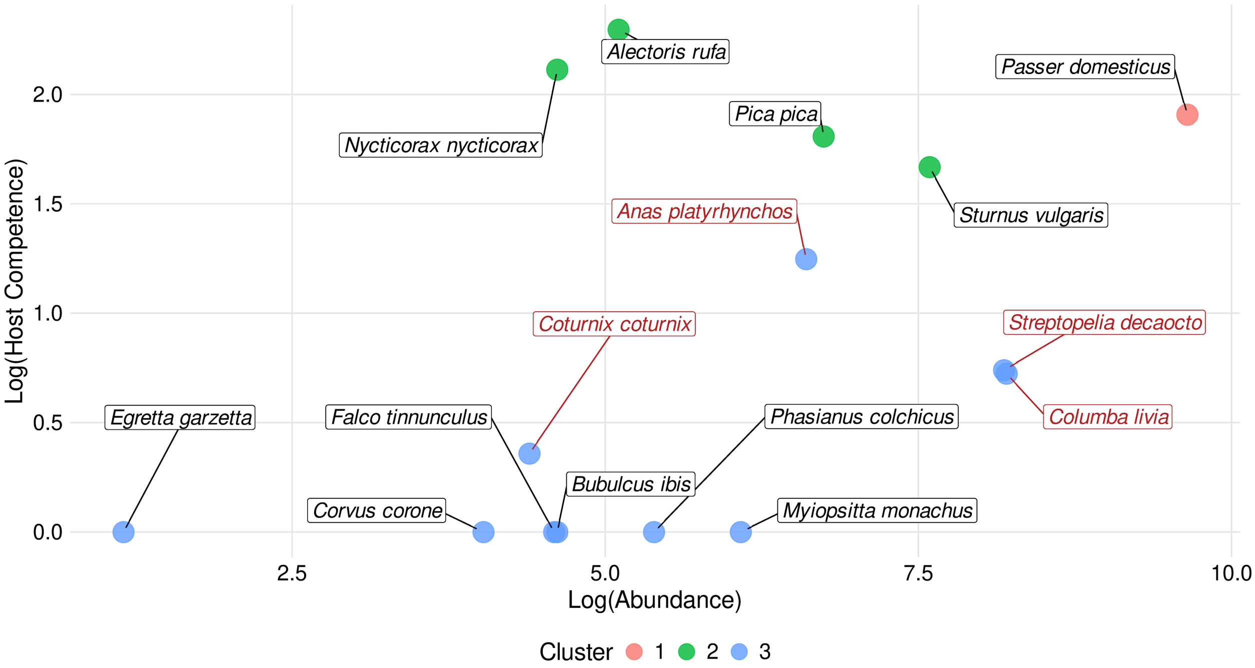

Fig B. Scatter plot illustrating the classification of avian species based on their log-transformed abundance and WNV host competence using the 10^4 PFU/ml threshold. Colors represent k-means clusters derived from a Principal Component Analysis (PCA), delineating three primary groups: 1) Species with high competence and abundance; 2) Species with high competence but lower abundance; 3) Species with low competence and/or low abundance, comprising the majority of non-reservoir species. Despite being reclassified as reservoirs under the 10^4 PFU/ml threshold, *Columba livia*, *Streptopelia decaocto*, and *Coturnix coturnix* cluster with non-reservoir species, further supporting the use of the 10^5 PFU/ml threshold for distinguishing biologically significant reservoirs. Similarly, *Anas platyrhynchos* clusters with non-reservoir species due to its host competence value being proximal to the threshold. However, given that its peak viremia exceeds both the 10^4 and 10^5 PFU/ml thresholds, it might be considered a moderately competent species for WNV transmission.

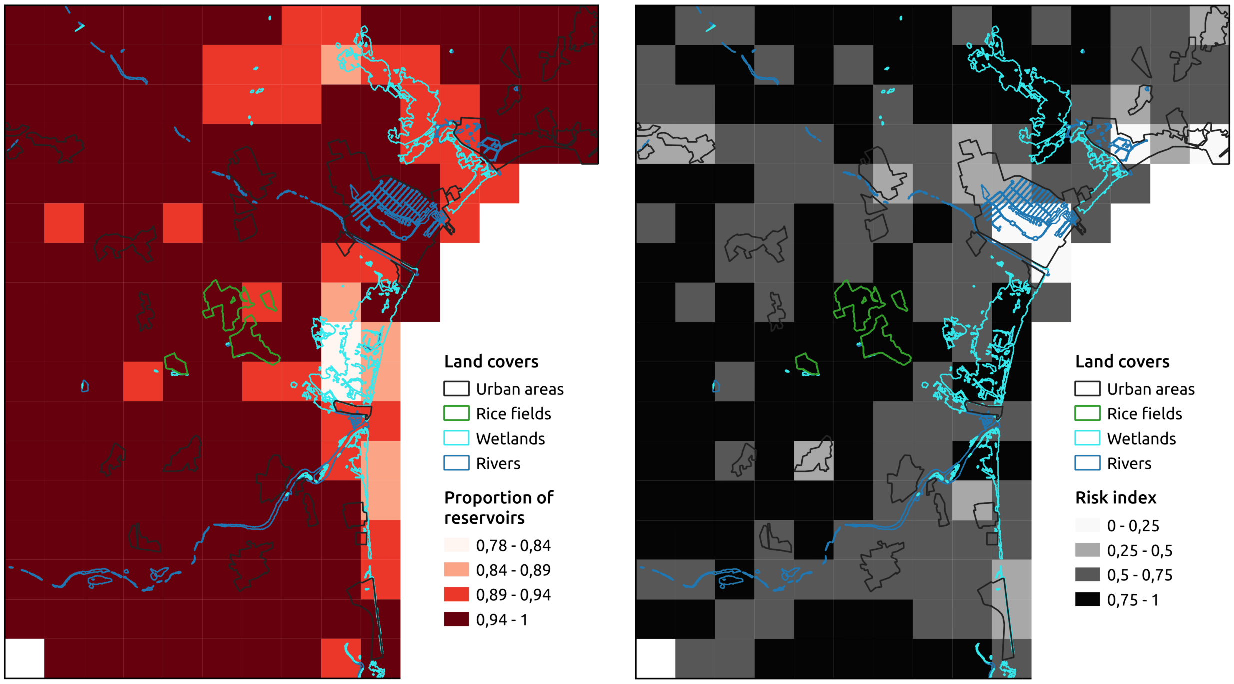

(a) Proportion of WNV reservoir birds

(b) WNV risk map

Fig C. Maps of the study area, divided into 1×1 km grid cells, showing the proportion of WNV reservoir birds and the WNV risk index using the 10^4 PFU/ml threshold. The visualization employs an equal-interval classification scheme. **(a)**: The proportion of WNV reservoir birds was recalculated using the adjusted infection threshold, leading to the reclassification of certain species. The resulting distribution exhibits a high degree of uniformity, with most grid cells showing elevated proportions of reservoir species. This pattern is likely driven by the inclusion of *Columba livia* and *Streptopelia decaocto* as reservoirs, given their widespread distribution and abundance. **(b)**: The WNV risk index was updated to incorporate the modified reservoir species classification. The number of grid cells with elevated risk values increased compared to the primary analysis. However, urban areas continue to exhibit lower risk scores due to the lower abundance of *Culex* vectors.
